# Disordered T cell-B cell interactions in autoantibody-positive inflammatory arthritis

**DOI:** 10.3389/fimmu.2022.1068399

**Published:** 2023-01-05

**Authors:** Amélie M. Julé, Ki Pui Lam, Maria Taylor, Kacie J. Hoyt, Kevin Wei, Maria Gutierrez-Arcelus, Siobhan M. Case, Mia Chandler, Margaret H. Chang, Ezra M. Cohen, Fatma Dedeoglu, Olha Halyabar, Jonathan Hausmann, Melissa M. Hazen, Erin Janssen, Jeffrey Lo, Mindy S. Lo, Esra Meidan, Jordan E. Roberts, Holly Wobma, Mary Beth F. Son, Robert P. Sundel, Pui Y. Lee, Peter T. Sage, Talal A. Chatila, Peter A. Nigrovic, Deepak A. Rao, Lauren A. Henderson

**Affiliations:** ^1^ Division of Immunology, Boston Children’s Hospital, Harvard Medical School, Boston, MA, United States; ^2^ Division of Rheumatology, Inflammation, and Immunity, Brigham and Women’s Hospital, Harvard Medical School, Boston, MA, United States; ^3^ Broad Institute of Massachusetts Institute of Technology (MIT) and Harvard, Cambridge, MA, United States; ^4^ Division of Rheumatology, Boston Medical Center, Boston University School of Medicine, Boston, MA, United States; ^5^ Transplantation Research Center, Renal Division, Brigham and Women’s Hospital, Harvard Medical School, Boston, MA, United States

**Keywords:** T cells, autoimmunity, autoantibodies, juvenile idiopathic arthritis, T peripheral helper cell, regulatory T (Treg) cell

## Abstract

T peripheral helper (Tph) cells, identified in the synovium of adults with seropositive rheumatoid arthritis, drive B cell maturation and antibody production in non-lymphoid tissues. We sought to determine if similarly dysregulated T cell-B cell interactions underlie another form of inflammatory arthritis, juvenile oligoarthritis (oligo JIA). Clonally expanded Tph cells able to promote B cell antibody production preferentially accumulated in the synovial fluid (SF) of oligo JIA patients with antinuclear antibodies (ANA) compared to autoantibody-negative patients. Single-cell transcriptomics enabled further definition of the Tph gene signature in inflamed tissues and showed that Tph cells from ANA-positive patients upregulated genes associated with B cell help to a greater extent than patients without autoantibodies. T cells that co-expressed regulatory T and B cell-help factors were identified. The phenotype of these Tph-like Treg cells suggests an ability to restrain T cell-B cell interactions in tissues. Our findings support the central role of disordered T cell-help to B cells in autoantibody-positive arthritides.

## 1 Introduction

B cell maturation and antibody production in secondary lymphoid organs is tightly regulated by the T follicular helper (Tfh) and T follicular regulatory (Tfr) axis. Tfh cells traffic to the lymphoid follicle *via* detection of the chemoattractant CXCL13 by their surface receptor CXCR5 (1–3). Once in the follicle, Tfh cells upregulate the transcription factor BCL6 and engage a gene expression profile that promotes B cell helper function (4–7). Tfh cells express factors (PD-1, ICOS, IL-21, CXCL13) that facilitate germinal center (GC) reactions and result in high affinity B cell maturation, leading to the generation of memory or antibody-secreting B cells (8–16). The germinal center reaction is restrained by a T cell subset with mixed features of regulatory T (Treg) and Tfh cells, known as Tfr cells (17–21). Tfr cells alter the affinity of foreign antigen specific antibodies (22). In addition, the Tfr population represents an important immune check-point to prevent autoreactive antibody-producing plasma cells from exiting the B cell follicle (21).

While interactions among B, Tfh, and Tfr cells are well characterized in lymphoid organs, less is understood about the interplay between these populations in peripheral tissues. T-B cell aggregates exist in inflamed tissues such as the synovium of adults and children with inflammatory arthritis, suggesting that T cells provide help to B cells in the periphery (23, 24). B cells from tissues with such ectopic lymphoid structures display features consistent with the GC reaction, including a memory phenotype and mutated B cell receptor (25). T peripheral helper (Tph) cells have recently been identified as a T cell subset that can promote B cell infiltration and maturation in inflamed tissues (9). Like Tfh cells, Tph cells express many factors associated with B cell helper function: PD-1, ICOS, IL-21, CXCL13 (9). Unlike Tfh cells that express the lymphoid trafficking receptor CXCR5, Tph cells display other receptors that allow entry into peripheral tissues (1, 2, 9). Further, Tph cells do not express high levels of BCL6, the lineage-defining transcription factor of Tfh cells. Thus, Tph cells represent a unique T cell population defined by a transcriptional program distinct from Tfh cells that allows these cells to reside in tissues. While Tfr cells prevent pathologic autoantibody production in secondary lymphoid organs, a corresponding population of Tregs with the capacity to inhibit Tph-B cell interactions in inflamed tissues has not been identified.

Pathogenic Tph cells were originally observed in the synovial tissue of patients with seropositive rheumatoid arthritis (RA), a form of inflammatory arthritis that is characterized by formation of synovial lymphoid aggregates and production of autoantibodies (9). We sought to determine if Tph cells may play a role in the pathogenesis of other types of arthritis that differ from seropositive RA. Notably, there exists a form of chronic inflammatory arthritis that is unique to children and characterized by limited joint involvement (<5 joints), young age at disease onset (<6 years), female predominance, and high risk for uveitis (26–28). Children with this type of early-onset, oligoarticular juvenile idiopathic arthritis (oligo JIA) are largely antinuclear antibody (ANA) positive but are negative for the antibodies associated with seropositive RA (rheumatoid factor and cyclic citrullinated peptide) (26, 27, 29). Interestingly, these patients display a B cell gene signature in whole blood and increased frequencies of circulating switched memory B cells that are not found in older children with JIA (30–33). Compared to ANA-negative JIA patients, synovial tissues from those with ANA positivity are more likely to demonstrate signs of lymphoid organization, including T-B cell aggregates and germinal center reactions (23). Previously, we conducted single-cell RNA sequencing (scRNA-seq) of synovial fluid CD4^+^ T cells from two patients with oligo JIA and identified a subset of T cells with B cell-helper features, which were more frequent in an ANA^+^ child (34). Fisher et al. has documented an enrichment in IL-21 producing cells in the joints of ANA^+^ JIA patients (35). These findings in early-onset and ANA^+^ oligo JIA suggest dysregulated B cell responses and a potential role for Tph cells in this form of inflammatory arthritis.

To further our understanding of Tph-B cell interactions in autoantibody-positive inflammatory arthritis, we performed a comprehensive analysis of T and B cells from the joints of children with ANA^+^ and ANA^-^ oligo JIA. Through protein-level characterization and in-depth single-cell transcriptomics, we show that Tph cells are enriched in children with ANA^+^ oligo JIA and confirm their capacity to promote B cell maturation *in vitro*. These findings further clarify the underlying biology that defines early-onset and autoantibody-positive arthritis in children. Owing to the presence of Tph cells in ANA^+^ JIA joints, we hypothesized that a subset of Tregs with the ability to counteract the activity of Tph cells might also exist in peripheral tissues, thereby exerting similar functions as Tfr cells in lymphoid organs. Indeed, our studies identified a Treg population with some of these characteristics.

## 2 Results

### 2.1 Patients and samples

The flow cytometry analyses and functional assays performed as part of this study included a total of 28 oligo JIA patients, defined by International League of Associations for Rheumatology (ILAR) criteria (**Table 1**; **Table S1**) (29). Fifteen patients were ANA^+^, defined as a detectable ANA titer at any time during the disease course. Five pediatric and 11 adult controls also contributed peripheral blood samples.

**Table 1:** Clinical characteristics of study participants.

	*ANA^-^ oligo JIA*	*ANA^+^ oligo JIA*
**Sex**	11 Female, 2 Male	14 Female, 1 Male
**Age (years) at onset**	5.1 ± 2.4 (2 – 11)	5.1 ± 3.2 (2 – 13)
**<6 years at onset**	8 subjects	10 subjects
**Active joints at onset**	1.3 ± 0.6 (1 – 3)	1.7 ± 1.0 (1 – 4)
**Age (years) at first sampling**	8.6 ± 3.1 (4 – 14.5)	9.1 ± 5.8 (2.5 – 20)
**Active joints at first sampling**	1.5 ± 0.7 (1 – 3)	1.9 ± 0.9 (1 – 4)
**New onset at first sampling**	5 subjects	5 subjects
**Disease course**	11 persistent, 2 extended, 1 unknown	12 persistent, 2 extended, 1 undifferentiated
**Medication at first sampling**	1 ADA, 1 LEF, 2 MTX	1 ADA, 1 ETA, 3 MTX

Summary values are mean ± standard deviation, with the range indicated in brackets. One patient from each ANA group provided samples at 2 distinct arthritic flares. See **Table S1** for additional information. Oligo, oligoarticular; JIA, juvenile idiopathic arthritis; ANA, antinuclear antibody; ADA, adalimumab; ETA, etanercept; LEF, leflunomide; MTX, methotrexate.

### 2.2 CD4^+^PD-1^hi^CXCR5^-^ T peripheral helper cells are enriched in the synovial fluid of autoantibody-positive oligo JIA patients.

**Figure 1:**
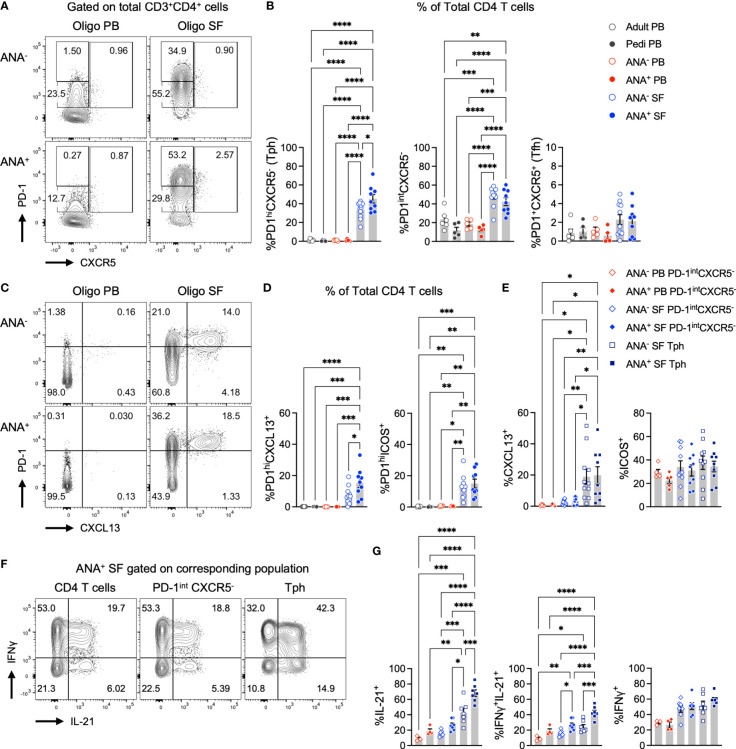
T peripheral helper cells expressing B cell help factors are enriched in the synovial fluid of autoantibody-positive oligo JIA patients. **(A)** Representative flow staining of B cell-helper CD4^+^ T cell populations in oligo JIA PB and SF, gated on total CD3^+^CD4^+^ T cells. In each experiment, the PD-1^hi^ gate was set by evaluating untstimulated PB samples from oligo JIA patients (often paired PB and SF sample from the same patient were used) and controls. This PD-1^hi^ gate was then applied to SF samples from oligo JIA patients evaluated during the same experiment. **(B)** Percent of Tph (PD-1^hi^CXCR5^-^), PD-1^int^CXCR5^+^, and Tfh (PD-1^+^CXCR5^+^) cells among CD4^+^ T cells in the SF of oligo JIA patients (ANA^-^: n=11, ANA^+^: n=9, blue circles), and in the PB of oligo JIA patients (ANA^-^: n=5, ANA^+^: n=5, red circles) and controls (pediatric: n=5, adult: n=6, black circles). **(C)** Representative flow staining of PD-1^hi^CXCL13^+^ T cells in oligo JIA PB and SF, gated on total CD3^+^CD4^+^ T cells. **(D)** Percent of PD-1^hi^CXCL13^+^ and PD-1^hi^ICOS^+^ cells among CD4^+^ T cells in the SF of oligo JIA patients (ANA^-^: n=10-11, ANA^+^: n=9), and in the PB of oligo JIA patients (ANA^-^: n=5, ANA^+^: n=5) and controls (pediatric: n=4-5, adult: n=6). **(E)** Percent of CXCL13^+^ and ICOS^+^ cells among CD4^+^PD-1^int^CXCR5^-^ and Tph subsets in the SF (ANA^-^: n=10-11, ANA^+^: n=9) and in the PB (ANA^-^: n=5, ANA^+^: n=5) of oligo JIA patients. **(F)** Representative flow staining of cytokine production in oligo JIA PB and SF, gated on CD4^+^ T cells and subpopulations. **(G)** Percent of IL-21^+^, IFNγ^+^ and dual IFNγ^+^IL-21^+^ cells among CD4^+^PD-1^int^CXCR5^-^ and Tph subsets in the SF (ANA^-^: n=7, ANA^+^: n=7) and in the PB (ANA^-^: n=3, ANA^+^: n=5) of oligo JIA patients. Oligo JIA, oligoarticular juvenile idiopathic arthritis; PB, peripheral blood; SF, synovial fluid; pedi, pediatric; ANA, antinuclear antibody; Tph, T peripheral helper cell; Tfh, T follicular helper cell. Summary data on bar graphs are mean ± standard error. P-value <0.05 (*); <0.01 (**), <0.001 (***), <0.0001 (****). Statistical testing: one-way ANOVA followed by multiple t-tests with Turkey correction.

Synovial fluid (SF) and peripheral blood (PB) samples from oligo JIA patients and controls were analyzed with flow cytometry to evaluate T cell populations known to provide help to B cells, notably T peripheral helper (Tph, CD4^+^PD-1^hi^CXCR5^-^) and T follicular helper (Tfh, CD4^+^PD-1^+^CXCR5^+^) cells (**Figure 1**; **S1**). While the frequency of Tph cells rarely exceeded 2% of CD4^+^ T cells in the PB of oligo JIA and control subjects, we found a dramatic enrichment of this population in the SF of oligo JIA patients (**Figures 1A, B**). In addition, Tph cells were significantly enriched in the SF of ANA^+^ oligo JIA (45.3 ± 4.6% of CD4^+^ T cells, mean ± standard error) compared to the SF of ANA^-^ oligo JIA patients (33.6 ± 2.6%, p-value = 0.03). Regardless of ANA status, there was no significant difference in the levels of Tfh cells between SF and PB, which remained low (<5%) in the vast majority of subjects.

To compare to Tph cells, we evaluated CD4^+^ T cells with intermediate PD-1 expression. CD4^+^PD-1^int^CXCR5^-^ T cells are observed at increased frequencies in patients with autoimmunity, but unlike Tph cells, they are not associated with measures of disease activity or end-organ involvement in autoimmune diseases such as systemic lupus erythematosus (SLE) (36). The frequency of CD4^+^PD-1^int^CXCR5^-^ cells was increased in the SF of oligo JIA patients compared to the PB of patients and controls, but no significant difference was detected between ANA^+^ (42.4 ± 4.2%) and ANA^-^ (48.3 ± 3.1%) SF samples (p-value = 0.75) (**Figures 1A, B**).

Taken together, these results suggest that Tph cells, and not Tfh cells, are the primary B cell-helper T cell subset in the joints of oligo JIA patients. Further, Tph cells are especially enriched in the SF of oligo JIA patients who are ANA^+^.

### 2.3 Tph cells in oligo JIA synovial fluid express markers of B cell-helper T cells

**Figure 2:**
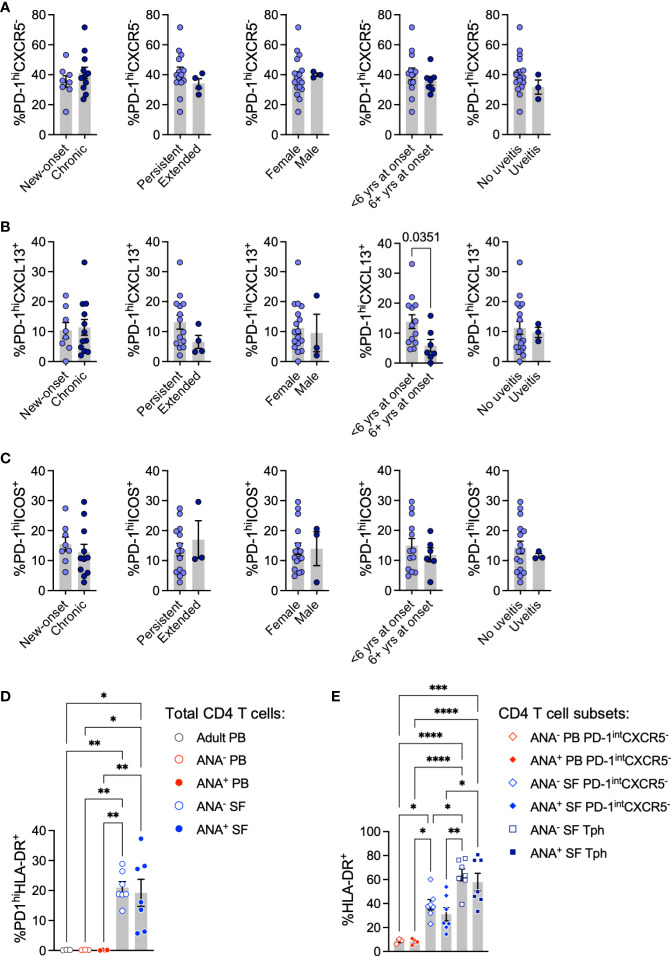
T peripheral helper cells in oligo JIA patients contrasted by disease characteristics. **(A-C)** Percent of Tph (CD4^+^PD-1^hi^CXCR5^-^, panel A), PD-1^hi^CXCL13^+^ (panel B), and PD-1^hi^ICOS^+^ **(C)** cells among total CD4^+^ T cells in the SF of oligo JIA patients (n=20) with varied disease characteristics. **(D)** Percent of PD-1^hi^HLA-DR^+^ cells among CD4^+^ T cells in the SF of oligo JIA patients (ANA^-^: n=7, ANA^+^: n=7, blue circles), and in the PB of oligo JIA patients (ANA^-^: n=3, ANA^+^: n=4, red circles) and adult controls (n=3, black circle). **(E)** Percent of HLA-DR^+^ cells among CD4^+^PD-1^int^CXCR5^-^ and Tph subsets in the SF (ANA^-^: n=7, ANA^+^: n=7) and in the PB (ANA^-^: n=3, ANA^+^: n=4) of oligo JIA patients. yrs, years; PB, peripheral blood; SF, synovial fluid; ANA, antinuclear autoantibody; Tph, T peripheral helper. Summary data on bar graphs are mean ± standard error. P-value <0.05 (*); <0.01 (**), <0.001 (***), <0.0001 (****). Statistical testing: **(A-C)** Two-tailed Student’s T-test; **(D-E)** one-way ANOVA followed by multiple t-tests with Turkey correction.

PD-1 expression in T cells from an inflammatory environment may indicate exhaustion or the capacity to promote B cell maturation. To determine whether Tph cells in oligo JIA SF present characteristics typical of B cell-helper T cells, we assessed the expression of markers that promote the B cell chemotactic response (CXCL13) and T-B cell interactions in GCs (ICOS) (3, 37, 38). Following stimulation with CD3/CD28-coated beads, the SF of oligo JIA patients showed a significant enrichment in CD4^+^PD-1^hi^CXCL13^+^ T cells and in CD4^+^PD-1^hi^ICOS^+^ T cells, which were absent in PB (**Figures 1C, D**; **S2**). The frequency of CD4^+^PD-1^hi^CXCL13^+^ T cells was significantly enriched in ANA^+^ SF (15.5 ± 3.1%) compared to ANA^-^ SF (7.4 ± 1.6%, p-value = 0.02). When gating on different sub-populations of CD4^+^ T cells, we confirmed that CXCL13 secretion was exclusive to SF Tph cells (**Figure 1E**). In contrast, ICOS was found in SF Tph but also in SF CD4^+^PD-1^int^CXCR5^-^ and PB CD4^+^PD-1^int^CXCR5^-^ T cells. ANA positivity in oligo JIA is most common in children with early-onset disease (26, 27). Interestingly, CD4^+^PD-1^hi^CXCL13^+^ T cells were also significantly increased in the SF of oligo JIA patients with disease onset at or before 6 years of age (13.8 ± 2.3%) compared to those who were older at the initial presentation of disease (5.8 ± 2.1%, p-value = 0.04) (**Figure 2**). There was a trend towards lower frequencies of CXCL13^+^ Tph cells in the small number of evaluated patients with extended oligo JIA (n=4); however, this did not reach statistical significance (p-value = 0.17).

We then assessed cytokine production in CD4^+^ T cell subsets from oligo JIA patients and controls, focusing on IL-21 due to its role in promoting B cell survival and maturation (39, 40). IL-21^+^ cells were largely enriched in oligo JIA SF Tph cells (CD4^+^PD-1^hi^CXCR5^-^) compared to other CD4^+^ T cell populations (PD-1^int^CXCR5^-^) in the joint or blood (**Figures 1F, G**). IL-21^+^ Tph cells were further enriched in ANA^+^ SF (68.1 ± 4.2%) compared to ANA^-^ SF (42.8 ± 5.6%, p-value = 0.0003). IFNγ^+^IL-21^+^ dual expressing cells were also most enriched in SF Tph cells. By contrast, the fraction of IFNγ^+^ cells did not differ between SF PD-1^int^CXCR5^-^ and SF Tph cells, suggesting that both populations contribute to IFNγ secretion in oligo JIA SF.

Finally, we assessed the expression of HLA-DR, a marker of activation associated with a Tph cell phenotype in RA (9). As previously described, we found that Tph cells in oligo JIA SF co-express HLA-DR to a greater extent than CD4^+^PD-1^int^CXCR5^-^ T cells; however, we found no significant difference in the frequency of HLA-DR^+^ Tph cells between ANA^+^ and ANA^-^ patients (**Figures 2D, E**) (41).

Altogether, these studies show that CD4^+^PD-1^hi^CXCR5^-^ (Tph) cells in oligo JIA SF express effector molecules that provide help to B cells, notably CXCL13 and IL-21. Further, both CXCL13 and IL-21 were most frequently expressed by SF Tph in ANA^+^ compared to ANA^-^ patients. This effector protein expression profile supports the hypothesis that PD-1 expression by Tph cells in oligo JIA does not simply reflect activation or exhaustion in the inflammatory environment of the arthritic joint.

### 2.4 Synovial fluid Tph cells promote the differentiation of memory B cells into plasmablasts

**Figure 3:**
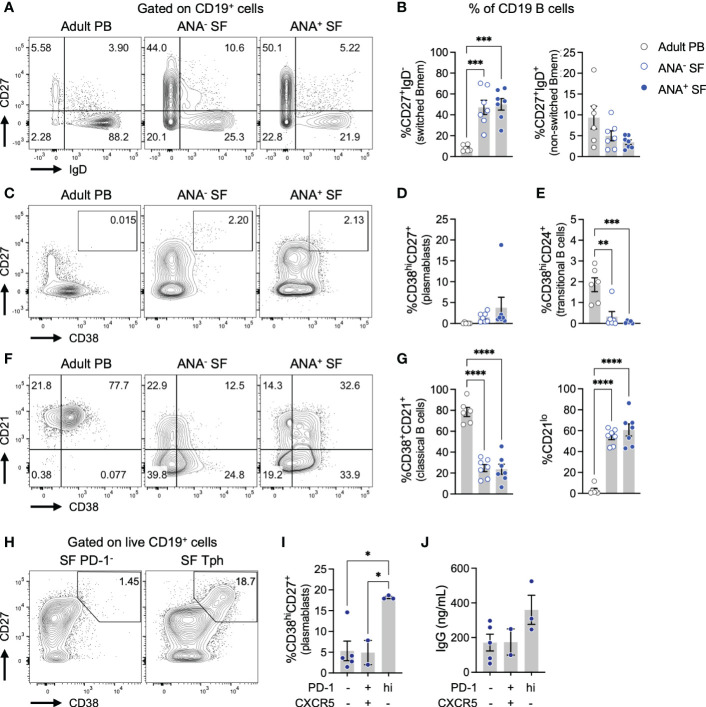
Tph cells in oligo JIA synovial fluid promote the differentiation of memory B cells into plasmablasts. **(A)** Representative flow staining of memory B (Bmem) cell populations, gated on total CD19^+^ B cells. **(B)** Percent of switched (CD27^+^IgD^-^) and unswitched (CD27^+^IgD^+^) Bmem among CD19^+^ B cells in the SF of oligo JIA patients (ANA^-^: n=7, ANA^+^: n=7, blue circles) and in the PB of adult controls (n=6, black circles). **(C)** Representative flow staining of CD38^hi^CD27^+^ plasmablasts, gated on total CD19^+^ B cells. **(D)** Percent of plasmablasts (CD38^hi^CD27^+^) among CD19^+^ B cells in the SF of oligo JIA patients (ANA^-^: n=7, ANA^+^: n=7) and in the PB of adult controls (n=6). **(E)** Percent of transitional B cells (CD38^hi^CD24^+^) among CD19^+^ B cells in the SF of oligo JIA patients (ANA^-^: n=7, ANA^+^: n=7) and in the PB of adult controls (n=6). **(F)** Representative flow staining of classical and CD21^lo^ B cell populations, gated on total CD19^+^ B cells. **(G)** Percent of classical (CD38^+^CD21^+^) and CD21^lo^ B cells among CD19^+^ B cells in the SF of oligo JIA patients (ANA^-^: n=7, ANA^+^: n=7), and in the PB of adult controls (n=6). **(H)** Representative staining of a co-culture of the indicated population of oligo JIA SF CD4^+^ T cells with PB Bmem cells from a third party control after 5 days of incubation in the presence of SEB (1 μg/mL), gated on total CD19^+^ B cells. **(I)** Percent of plasmablasts (CD38^hi^CD27^+^) among CD19^+^ B cells in co-cultures with the corresponding SF CD4^+^ T cell population (3 independent experiments). **(J)** Concentration of IgG detected in the supernatant of B cell co-cultured with the corresponding SF CD4^+^ T cell population (3 independent experiments). Oligo JIA, oligoarticular juvenile idiopathic arthritis; PB, peripheral blood; SF, synovial fluid; ANA, antinuclear antibody; Tph, T peripheral helper cell; SEB, staphylococcal enterotoxin **(B)** Summary data on bar graphs are mean ± standard error. P-value <0.05 (*); <0.01 (**), <0.001 (***), <0.0001 (****). Statistical testing: one-way ANOVA followed by multiple t-tests with Turkey correction.

We next leveraged flow cytometry to evaluate the phenotype of B cells in oligo JIA SF (**Figures 3A-G**; **S3**). Switched memory B cells (CD27^+^IgD^-^, Bmem) were significantly increased among CD19^+^ B cells in the SF of oligo JIA patients compared to the PB of controls (**Figures 3A, B**). Plasmablasts were not enriched in oligo JIA SF, regardless of ANA status (**Figures 3C, D**). However, CD19^+^ B cells in oligo JIA SF displayed unusual characteristics: transitional (CD38^hi^CD24^+^) and classical (CD38^+^CD21^+^) B cells were depleted in oligo JIA SF compared to control PB, while CD21^lo^ B cells, a potentially autoreactive subclass of B cells that has been identified in other autoimmune and immune dysregulatory conditions, were enriched (**Figures 3E-G**) (42, 43).

To assess the functionality of SF Tph cells, we studied their capacity to promote the differentiation of Bmem cells into antibody-producing plasmablasts *in vitro* (**Figures 3H-J**, **S3**). Different populations of CD4^+^ T cells were sorted from the SF of oligo JIA patients and co-cultured with Bmem cells (CD27^+^IgD^-^) from the PB of a third-party control. After 5 days of culture in the presence of the superantigen staphylococcal enterotoxin B (SEB), the frequency of CD27^+^CD38^hi^ plasmablasts was assessed. In co-cultures with double negative CD4^+^PD-1^-^CXCR5^-^ T cells or with Tfh cells from oligo JIA SF, the fraction of plasmablasts generally remained low (<5%). In contrast, co-culture of Bmem with SF Tph cells induced the production of plasmablasts with 18.2 ± 0.3% of CD27^+^CD38^hi^ detected on average (**Figures 3H, I**). Accordingly, the concentration of IgG trended higher in the supernatant of co-cultures with SF Tph cells compared to co-cultures with other SF CD4^+^ T cell populations, although this effect did not achieve statistical significance (**Figure 3J**).

Taken together, these results indicate that SF Tph cells are a functional population with the capacity to promote the differentiation of antibody-producing B cells.

### 2.5 The regulatory T cell compartment in oligo JIA synovial fluid encompasses cells with a Tph profile

**Figure 4:**
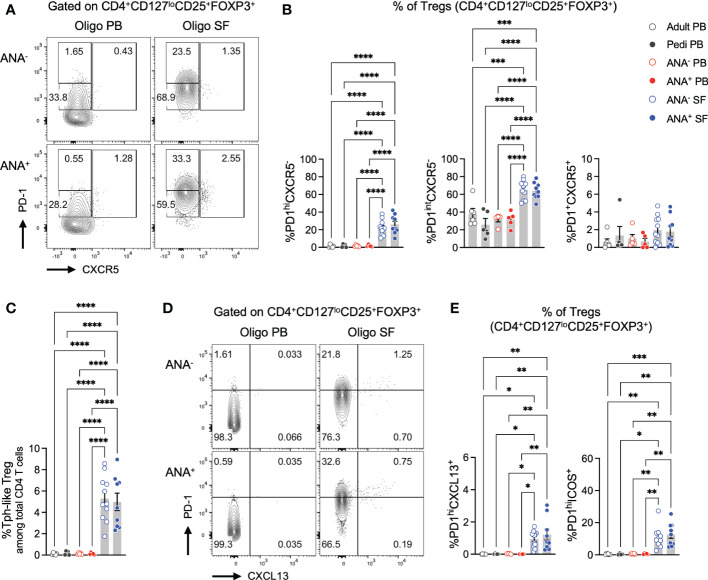
T regulatory cells expressing B cell help factors are enriched in oligo JIA synovial fluid. **(A)** Representative flow staining of B cell helper CD4^+^ T cell populations in oligo JIA PB and SF, gated on Tregs (CD3^+^CD4^+^CD127^lo^CD25^+^FOXP3^+^). In each experiment, the PD-1^hi^ gate was set by evaluating unstimulated Treg cells from PB (often paired PB and SF sample from the same patient were used) and controls. This PD-1^hi^ gate was then applied to SF samples from oligo JIA patients evaluated during the same experiment. **(B)** Percent of Tph-like Treg (PD-1^hi^CXCR5^-^), PD-1^int^CXCR5^+^, and Tfr (PD-1^+^CXCR5^+^) cells among Tregs in the SF of oligo JIA patients (ANA^-^: n=11, ANA^+^: n=9, blue circles), and in the PB of oligo JIA patients (ANA^-^: n=5, ANA^+^: n=5, red circles), and controls (pediatric: n=5, adult: n=6, black circles). **(C)** Percent of Tph-like Treg (CD4^+^CD127^lo^CD25^+^FOXP3^+^PD-1^hi^CXCR5^-^) cells among total CD3^+^CD4^+^ T cells. **(D)** Representative flow staining of PD-1^hi^CXCL13^+^ T cells in oligo JIA PB and SF, gated on Tregs. **(E)** Percent of PD-1^hi^CXCL13^+^ and PD-1^hi^ICOS^+^ cells among Tregs in the SF of oligo JIA patients (ANA^-^: n=10-11, ANA^+^: n=9), and in the PB of oligo JIA patients (ANA^-^: n=5, ANA^+^: n=5) and controls (pediatric: n=4-5, adult). Oligo JIA; oligoarticular juvenile idiopathic arthritis; PB, peripheral blood; SF, synovial fluid; ANA, antinuclear antibody; pedi, pediatric; Tfr, T follicular regulatory cell. Summary data on bar graphs are mean ± standard error. P-value <0.05 (*); <0.01 (**), <0.001 (***), <0.0001 (****). Statistical testing: one-way ANOVA followed by multiple t-tests with Turkey correction.

In lymphoid organs, the GC reaction is regulated in part by T follicular regulatory (Tfr) cells, a subset of CD4^+^ T cells that share features of regulatory T (Treg) and Tfh cells (17–21, 44). We sought to determine if a similar population of Treg cells with Tph features might exist in peripheral tissues such as the joints of children with oligo JIA. Flow cytometry studies demonstrated that PD-1^hi^CXCR5^-^ cells were observed within the SF Treg population (CD4^+^CD127^lo^CD25^+^FOXP3^+^ cells, **Figures 4A, B**; **S1**). PD-1^hi^CXCR5^-^ Tregs, hereafter referred to as Tph-like Tregs, were enriched in the SF of ANA^+^ and ANA^-^ patients compared to the PB of patients and controls. Overall, these Tph-like Tregs accounted for 5.2 ± 0.5% of SF CD4^+^ T cells and were found at similar frequencies in ANA^+^ and ANA^-^ samples (**Figure 4C**). Similar to our observations in total CD4^+^ T cells, PD-1^int^CXCR5^-^ cells were also enriched in oligo JIA SF Tregs compared to PB Tregs, while PD-1^+^CXCR5^+^ cells were not.

To confirm that PD-1^hi^CXCR5^-^ Tregs express markers associated with T-B cell interactions, we evaluated CXCL13 and ICOS expression in Tregs (**Figures 4D, E**, **S2**). PD-1^hi^CXCL13^+^ cells were not detected in PB Tregs of oligo JIA or control subjects but were modestly yet significantly enriched in ANA^+^ SF Tregs (1.2 ± 0.3%) and ANA^-^ SF Tregs (0.9 ± 0.2%). Similarly, frequencies of PD-1^hi^ICOS^+^ cells remained low in PB Tregs (<2%), and were significantly elevated in ANA^+^ SF Tregs (12.6 ± 2.4%) and ANA^-^ SF Tregs (10.9 ± 2.1%).

In total, these studies suggest the presence of a Treg population with Tph features that reside in inflammatory environments outside of lymphoid organs, including a subset that express the B cell help factors CXCL13 and ICOS.

### 2.6 Synovial fluid Tph and Tph-like Treg cells express similar markers and robust levels of *CXCL13* at the transcriptomic level

**Figure 5:**
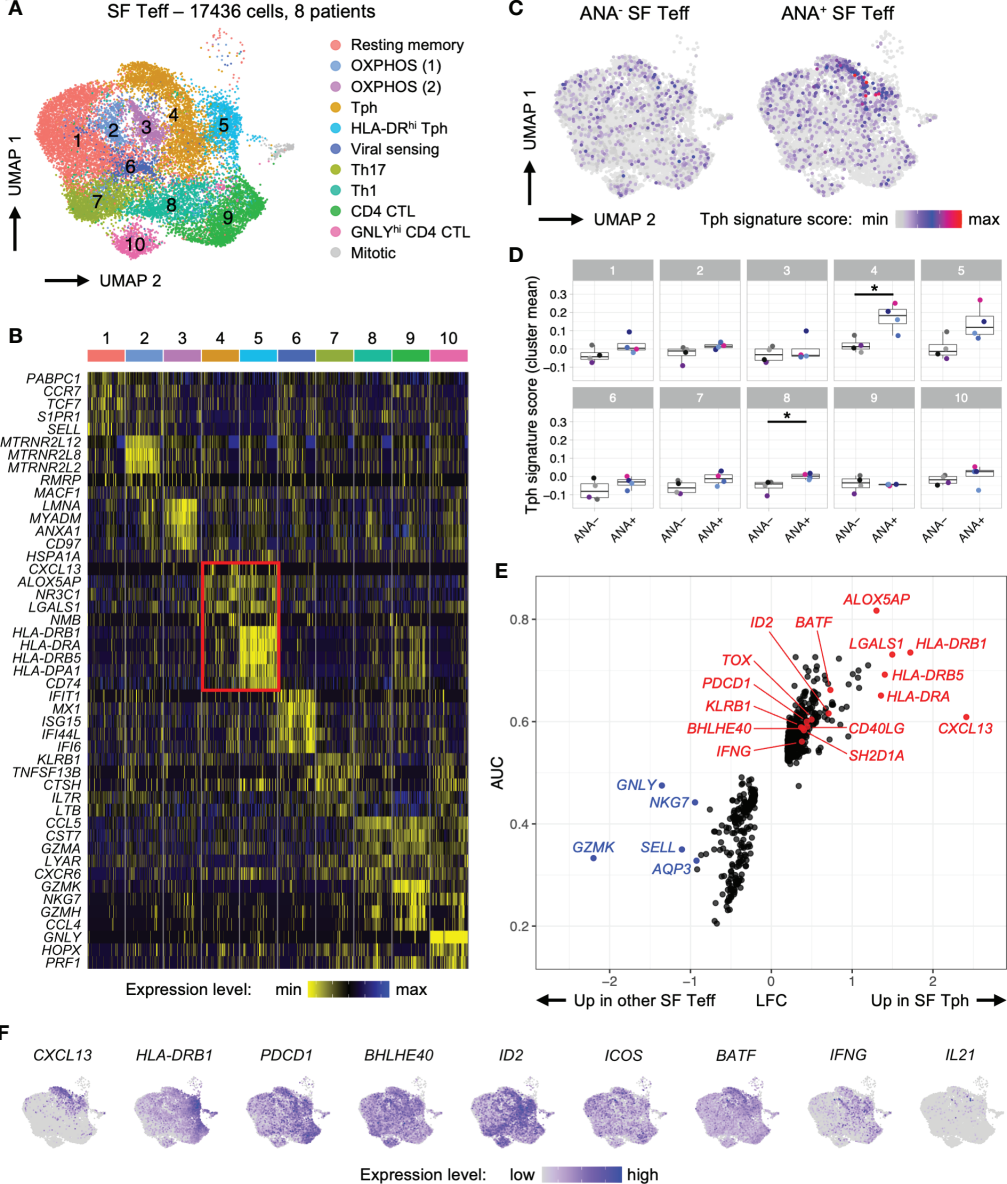
T peripheral helper cells in oligo JIA synovial fluid adopt a distinctive transcriptomic signature characterized by robust expression of CXCL13. Combined data from two previously published datasets of oligo JIA SF Teff (CD4^+^CD25^-^) cells studied with 10X Genomics (34, 45). **(A)** UMAP of SF Teffs from 8 oligo JIA patients, color-coded by cluster. **(B)** Heatmap of gene expression levels for the top 5 markers identified in each cluster using a Wilcoxon rank sum test, shown for 100 randomly selected cells from each cluster. **(C)** UMAP of SF Teffs split between ANA^-^ (9667 cells) and ANA^+^ (7769 cells) groups, color-coded with their Tph signature score. **(D)** Mean Tph signature score, averaged per patient and cluster. T-test comparisons of ANA^-^ versus ANA^+^ groups significant at a FDR of 0.05 are indicated with a star. **(E)** LFC versus AUC plot of differential expression analysis of SF Tph (Clusters 4 and 5) versus other SF Teffs (Clusters 1-3 and 6-10) run with Seurat v4 using the receiving operating characteristic (ROC) approach. **(F)** UMAP of SF Teffs showing expression levels of selected Tph genes in the top 95% percentile of the gene expression level distribution for each gene. Oligo JIA, oligoarticular juvenile idiopathic arthritis; SF, synovial fluid; ANA, antinuclear antibody; Teff, T effector cell; Tph, T peripheral helper cell; OXPHOS, oxidative phosphorylation; CTL, cytotoxic lymphocyte; UMAP, uniform manifold approximation and projection; AUC, area under the curve; LFC, log2 fold change; FDR, false discovery rate.

**Figure 6:**
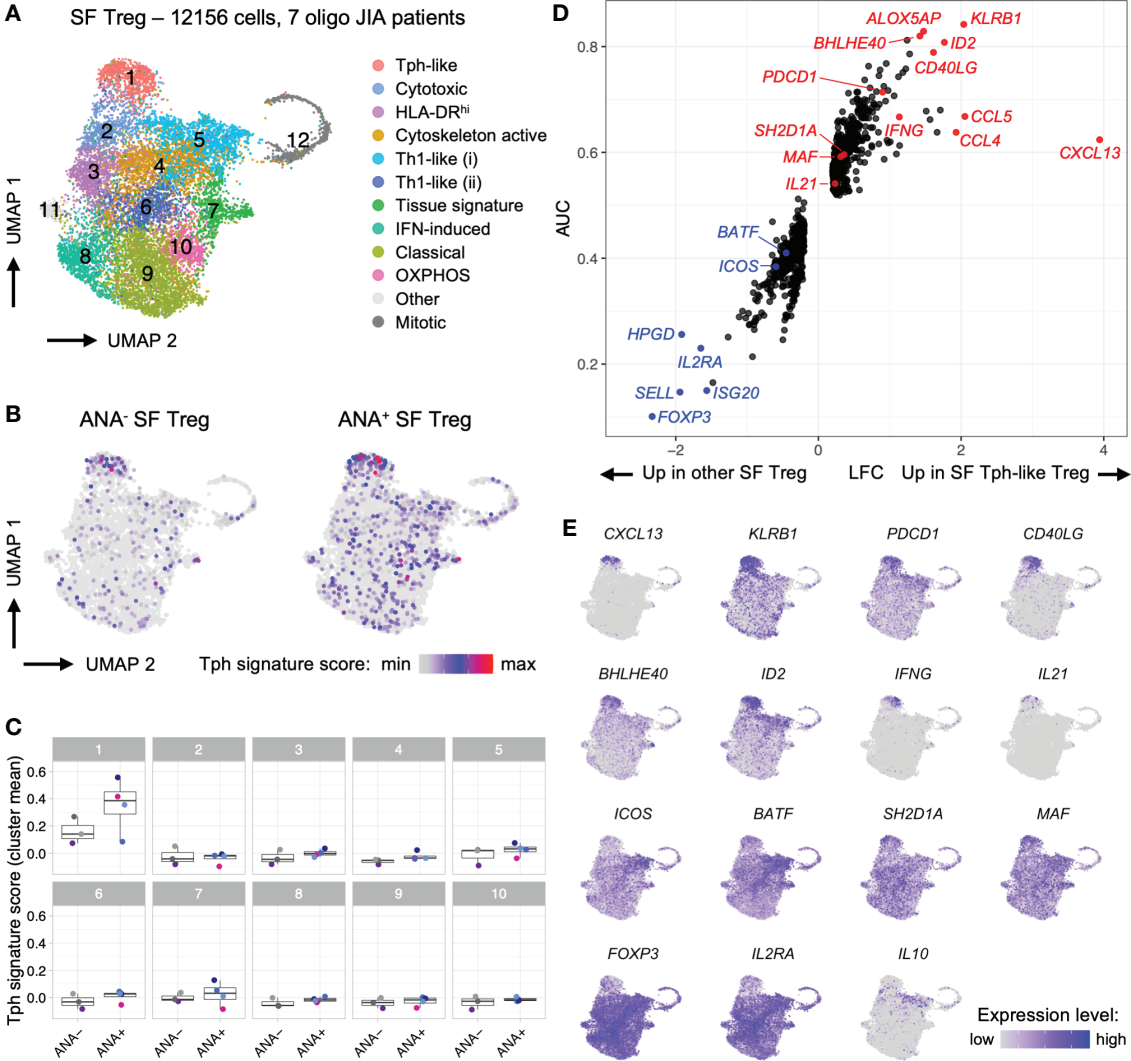
T regulatory cells in oligo JIA synovial fluid adopt a distinctive transcriptomic signature characterized by robust expression of CXCL13. Combined data from two previously published datasets of oligo JIA SF Treg (CD4^+^CD127^lo^CD25^+^) cells studied with 10X Genomics (34, 45). **(A)** UMAP of SF Tregs from 7 oligo JIA patients, color-coded by cluster. **(B)** UMAP of SF Tregs split between ANA^-^ (4550 cells) and ANA^+^ (7606 cells) groups, color-coded with their Tph signature score. **(C)** Mean Tph signature score, averaged per patient and cluster. Multiple T-test comparisons of ANA^-^ versus ANA^+^ groups revealed no significant difference after FDR correction. **(D)** LFC versus AUC plot of differential expression analysis of SF Tph-like Tregs (Cluster 1) versus other SF Tregs (Clusters 2-11) run with Seurat v4 using the receiving operating characteristic (ROC) approach. **(E)** UMAP of SF Tregs showing expression levels of selected Tph-like Treg genes in the top 95% percentile of the gene expression level distribution for each gene. Oligo JIA; oligoarticular juvenile idiopathic arthritis; SF, synovial fluid; Treg, T regulatory cell; Tph, T peripheral helper cell; ANA, antinuclear antibody; OXPHOS, oxidative phosphorylation; UMAP, uniform manifold approximation and projection; AUC, area under the curve; LFC, log2 fold change; FDR, false discovery rate.

**Figure 7:**
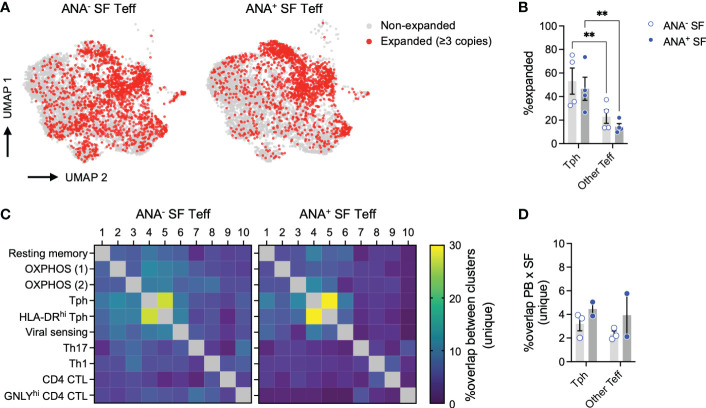
T peripheral helper cells in oligo JIA SF are clonally expanded. **(A)** UMAP of SF Teffs with known TCR alpha and beta chains from 8 oligo JIA patients, highlighting expanded clonotypes. **(B)** Percent of expanded clonotypes among total SF Tph (Clusters 4 and 5) or other SF Teff (Clusters 1-3 and 6-10) cells. **(C)** Heatmap showing the percent of overlap in unique clonotypes between each pair of clusters identified in SF Teffs, split by ANA status. **(D)** Percent of overlap in unique clonotypes between total PB Teff and SF Tph (Clusters 4 and 5) or other SF Teff (Clusters 1-3 and 6-10) cells. Oligo JIA; oligoarticular juvenile idiopathic arthritis; SF, synovial fluid; PB, peripheral blood; Teff, effector T cell; Tph, T peripheral helper cell; ANA, antinuclear antibody; UMAP, uniform manifold approximation and projection; OXPHOS, oxidative phosphorylation; CTL, cytotoxic lymphocyte. Summary data on bar graphs are mean ± standard error. P-value < 0.01 (**), Statistical testing: two-way ANOVA followed by t-tests on Tph versus other Teff for each subgroup (ANA^-^ and ANA^+^) with Šídák correction.

To complement our flow cytometry studies and validate key markers of Tph and Tph-like Tregs, we studied the transcriptomic profile of SF Teff (CD4^+^CD25^-^) and SF Treg (CD4^+^CD127^lo^CD25^+^) cells at the single-cell level. We combined and re-analyzed 2 previously published datasets, enabling us to assess 17,436 SF Teffs and 12,156 SF Tregs from 4 ANA^+^ and 4 ANA^-^ oligo JIA patients (**Figures 5**-**7**, **S4**) (34, 45).

The combined analysis of 8 oligo JIA patients revealed similar SF Teff clusters as identified in Julé et al. where only 2 patients were studied while also providing a much greater number of transcripts to evaluate (**Figures 5A, B**; **Supplemental File S1**) (34). Notably, 2 clusters (4 and 5) expressed robust levels of the B cell help factor *CXCL13* alongside activation markers typical of B cell-helper T cells, including *PDCD1* and *CD40LG* (**Supplemental File S1**). In addition, the transcription factor *BATF*, which is important for the differentiation of B cell-helper T cells, was increased in these clusters (**Figures 5E, F**) (46). Thus, clusters 4 and 5 were delineated as Tph cells. Cluster 5 was further characterized by elevated levels of *HLA-DR* transcripts (**Figure 5B**). Both Tph clusters encompassed cells from the SF of ANA^+^ and ANA^-^ patients (**Figures S4A, B**); however, Tph cells originating from ANA^+^ SF samples showed substantially higher scores for the Tph transcriptomic signature as described in RA (**Figures 5C, D**) (47). Interestingly, the transcriptomic signature of Tph cells in oligo JIA SF differed from the transcriptomic signature of Tfh cells (**Figure S4C**) (44).

To further delineate the transcriptomic signature of Tph cells in oligo JIA, we performed a differential gene expression analysis (DEA) of cells in Tph clusters versus cells in other Teff clusters (**Figures 5E, F**; **Supplemental File S1**). This DEA highlighted 482 significantly upregulated genes in oligo JIA SF Tph and confirmed the increased expression of multiple T cell activation markers in Tph cells, including HLA-DR genes, *PDCD1*, *CD40LG* and *KLRB1* (encoding CD161). *CXCL13* appeared as the most upregulated gene in oligo JIA SF Tph cells. Other B cell help factors with increased expression in SF Tph cells included *BATF* and *SH2D1A/SAP*. In contrast, *ICOS* was not identified as a differentially expressed gene. Transcription factors typically expressed in Tfh or Tph cells, namely *BCL6* and *PRDM1*, were also not robustly expressed in oligo JIA SF Tph cells. However, our DEA highlighted other transcription factors that may play a role in shaping the transcriptomic landscape of Tph cells, such as *BHLHE40*. A putative central role for this basic helix-loop-helix transcription factor in oligo JIA SF Tph was supported by the simultaneous upregulation of *BHLHE40-AS1* and *ID2* in these cells, both of which might act as regulators of BHLHE40. In addition, *ALOX5AP*, which plays a role in leukotriene biosynthesis, was highly upregulated in Tph cells compared to other Teff populations in the joint.

We then ran a gene set enrichment analysis (GSEA) on the genes identified as significantly upregulated in oligo JIA SF Tph (**Figure S4D**). This GSEA showed that the transcriptomic signature of Tph cells in oligo JIA is enriched in genes associated with IFNγ signaling, antigen presentation and TCR signaling, suggestive of cytokine-producing, antigen-activated T cells. In the absence of stimulation, cytokine expression is barely detectable in 10X studies; therefore, we could not quantitatively assess IL-21 production by SF Tph at the transcriptomic level, although the few *IL21* expressing cells were primarily observed in Tph clusters (**Figure 5F**).

During the combined analysis of SF Tregs, a distinct cluster of Tph-like Tregs (Cluster 1) emerged from this re-analysis with a larger number of cells and patients (**Figure 6**; **Supplemental File S1**) (34, 45). The Tph transcriptomic signature derived from Zhang et al. was expressed at the highest level in cells falling within this cluster (**Figures 6B, C**) (47). DEA of Tph-like Tregs versus other Tregs confirmed that these cells in oligo JIA SF share key markers with Tph and other CD4^+^ T cells that help B cells, including *PDCD1*, *CD40LG*, *CXCL13*, *IL21*, *SH2D1A/SAP* and *MAF* (**Figures 6D, E**). *KLRB1* (encoding CD161), *ID2*, *BHLHE40*, and *ALOX5AP* also emerged as key markers of SF Tph-like Tregs. In contrast, Tph-like Tregs did not express elevated levels of *ICOS* or *BATF*. As has been demonstrated in Tfr cells in secondary lymphoid organs, Tph-like Tregs expressed lower levels of *FOXP3* and *IL2RA* than other SF Tregs but did not present transcriptomic features of induced Tregs (**Figure S4E**) (48). Furthermore, when directly contrasted to SF Teffs, Tph-like Tregs maintained high expression of key Treg associated genes. Among the top significant differentially expressed genes (DEGs), appeared *FOXP3* (average log2 fold change between SF Tph-like Tregs and total SF Teff LFC = 1.28), *IL2RA* (LFC = 1.31), *CTLA4* (LFC = 1.18) *TNFRSF18* (encoding GITR; LFC = 1.85), *TNFRSF4* (encoding OX40; LFC = 1.62), and *LAG3* (LFC = 1.50) (**Supplemental File S1**). These single-cell studies demonstrate a transcriptional program in a subset of Tregs consistent with B cell-helper T cells.

### 2.7 Tph cells are clonally expanded in oligo JIA synovial fluid

The combined SF Teff single-cell dataset included 11,604 cells with available T cell receptor (TCR) sequencing data for both the alpha and beta chains. Key repertoire metrics for each patient, including the number of total and unique clones, are detailed in **Table 2**. Compared to other SF Teff clusters, Tph clusters were significantly enriched in expanded clones (defined as clones with at least 3 copies across the dataset), regardless of ANA status (**Figures 7A, B**). SF Tph had a distinct clonotypic profile: while the 2 Tph clusters shared up to 30% of clonotypes with each other; they shared only few with other Teff clusters (**Figure 7C**). A small fraction (2-6%) of SF Tph clonotypes were also found in the blood (**Figure 7D**). Other SF Teff cells showed similar levels of overlap with PB Teff, suggesting that Tph and other Teff cells in oligo JIA SF recirculate in the blood to a similar extent (**Figure 7D**). In summary, Tph cells in the arthritic joint are expanded and demonstrate a TCR repertoire that is distinct from other SF effector T cells.

**Table 2:** Key TCR repertoire metrics from single-cell transcriptomics analysis of SF Teffs.

Patient	Total SF Teff	Unique SF Teff	Max prod freq	Median prod freq (Top 100)
JIA1	1610	1330	0.75%	0.19%
JIA2*	2002	1213	2.60%	0.25%
p1	1882	1535	0.85%	0.16%
p2*	1414	1090	1.13%	0.21%
p3*	1227	1062	0.73%	0.16%
p4*	1530	1199	0.78%	0.20%
p5	1348	828	12.8%	0.22%
p6	1209	613	9.59%	0.21%

*ANA^+^ oligo JIA patient. Patient IDs reflect those used in the study from which the 10X dataset was extracted (34, 45). Clonal counts and frequencies in the Teff repertoire were computed before exclusion of the contaminating Treg cluster identified in clustering analysis. prod freq, productive frequency. TCR, T cell receptor; SF, synovial fluid, Teff, effector T cell; JIA, juvenile idiopathic arthritis; prod, productive; freq, frequency.

## 3 Discussion

In this study, we show that Tph cells accumulate in the joints of oligo JIA patients and are enriched in the SF of children with autoantibodies (ANA^+^ patients). At the transcriptomic and protein level, SF Tph cells from ANA^+^ patient more frequently express factors known to promote T cell-help to B cells and the GC reaction. *In vitro* assays support the functionality of oligo JIA SF Tph cells in promoting the differentiation of B cells into antibody-producing plasmablasts. Further, Tph cells are the most clonally expanded CD4^+^ T cell population in oligo JIA SF, supporting the importance of this T cell subset in mediating immune responses in the joint. Our work also uncovered a subset of Treg cells found in SF with features mirroring Tph cells. Building on studies in RA, these findings support a role for Tph cells in the pathogenesis of autoantibody-positive arthritis.

Tph cells were originally identified by Rao et al. in the joints of patients with RA and have since been described in other autoimmune conditions driven by autoantibodies (9, 36, 49). In RA, marked expansion of Tph cells was specific to individuals with seropositive disease (patients with rheumatoid factor, RF and cyclic citrullinated peptide, CCP autoantibodies). We also find preferential enrichment of Tph cells in children with the ANA^+^ form of oligo JIA. While seropositive RA and oligo JIA are both forms of chronic inflammatory arthritis, they are distinct diseases with unique clinical features and varying autoantibody profiles (RF and CCP in RA and ANA in oligo JIA). The discovery of Tph cells in both conditions supports the hypothesis that autoantibody-positive arthritides share a final common pathway of dysregulated T-B cell interactions in the joint.

Tph cells in oligo JIA SF share many features with those in RA, including expression of PD-1, CXCL13, ICOS, IL-21, and HLA-DR but not CXCR5 at the protein level (9). By flow cytometry, we find that IL-21, and even more so, CXCL13, are specific to SF Tph cells, while ICOS and HLA-DR are found in Tph and other activated CD4^+^ T cell subsets alike. At the transcriptomic level, Tph cells from RA and oligo JIA have a mutual gene expression program characterized by upregulation of multiple genes associated with B cell help such as *PDCD1*, *CXCL13*, *CD40LG*, *SH2D1A/SAP* and *BATF* (9, 47, 50). One notable exception is *PRDM1* (encoding BLIMP1), which is expressed in RA Tph cells but was not significantly associated with a Tph phenotype in our scRNA-seq dataset (9). As in RA, the transcriptome of Tph cells from oligo JIA patients is distinct from the transcriptome of Tfh cells that provide help to B cells in lymph nodes, despite overlap of some B cell help genes (4–7, 44). In addition, we and others show that Tph cells demonstrate features of Th1 skewing, which are less common in Tfh cells (9, 51, 52). These findings highlight the unique gene expression program adopted by Tph cells to function in peripheral tissues.

While Tph cells are present in ANA^+^ and ANA^-^ forms of oligo JIA, they are significant enriched and demonstrated features of excessive activation, at both the protein and gene expression level, in autoantibody-positive patients. Compared to ANA^-^ patients, ANA^+^ oligo JIA patients have higher frequencies of CXCL13^+^, IL-21^+^ and, to a lesser extent, HLA-DR^+^ Tph cells. Further, CXCL13^+^ Tph cells are more frequent in children with early-onset disease, a clinical characteristic that tracks with ANA-positivity in patients with JIA. Given the important role of CXCL13 and IL-21 in promoting GC reactions, our findings suggest that these markers define a population of highly activated and potentially pathogenic Tph cells in autoantibody-positive arthritides (3, 8, 13, 14, 38). At the gene expression level, ANA^+^ oligo JIA patients present important differences in the transcriptomic profile of their SF T cells, with an enhanced Tph gene signature, suggestive of robust B cell help activity. At the single-cell level, CD4^+^ T cells that adopt the Tph gene signature are significantly more clonally expanded than other effector T cell subsets. In addition, clonally expanded T cells preferentially concentrate in the Tph compartment for ANA^+^ positive patients. Further, the TCR repertoire of Tph cells is unrelated to other T cell populations in the joint, suggesting a distinct origin for SF Tph cells. Thus, Tph cells from patients with ANA^+^ arthritis are clonally expanded and display features suggestive of an enhanced capacity to promote B cell maturation and antibody production in inflamed tissues.

Beyond Tph cells, our study uncovered a subset of Tph-like cells within the SF Treg population. By flow cytometry, these Tph-like Tregs are found in the pool of CD4^+^CD127^lo^CD25^+^FOXP3^+^ (Treg) cells and express high levels of PD-1. On average, over 20% of Tregs in SF are PD-1^hi^ in oligo JIA, and a fraction of these cells are positive for the B cell help factors CXCL13 and ICOS. Interestingly, the frequency of Tph-like Tregs, as measured by flow cytometry, did not differ in ANA^+^ compared to ANA^-^ oligo JIA patients. This may suggest that pathogenic Tph cells drive disease in autoantibody-positive oligo JIA rather than Treg dysfunction. Compared to other Treg subsets in oligo JIA SF, this Treg subset adopted a Tph gene expression signature with marked upregulation of *CXCL13* in particular, alongside *PDCD1*, *CD40L*, *IL-21*, *SH2D1A/SAP* and *MAF*. Previously, we showed that Tregs in oligo JIA SF adopt a Th1-like phenotype while remaining stably committed to the Treg lineage, and identified a small subset of IFNγ expressing Tregs by flow (34). Similarly, Tph-like Tregs demonstrated some characteristics of Th1 cells, including expression of IFNγ.

At the transcriptomic level, Tph-like Tregs express lower levels of Treg-associated genes like *FOXP3*, *IL2RA* and *IKZF2* (encoding Helios) than other Treg subsets. This raises the concern that some Teff cells, and especially Tph cells, may have been accidently captured as Tregs in the sorting strategy that was used for the scRNA-seq studies and relied on cell surface markers. Several of our findings support the regulatory T cell origin of these cells. First, Tregs with features of T cell-helper B cells were identified not only in scRNA-seq but also by flow cytometry, where intracellular detection of FOXP3 could be used to define this population. Second, compared to other SF Teffs, Tph-like Tregs maintained significantly elevated expression levels of Treg-associated genes, indicative of a regulatory T cell identity. Finally, the Tph-like Treg profile we describe here is consistent with observations in Tfr cells, which co-opt portions of the Tfh transcriptional program to gain access to the lymphoid follicle and enhance B cell suppressor functions (17, 18, 44). Some aspects of Tfh gene expression are antagonistic to Treg cells, resulting in downregulation of some key Treg genes in Tfr cells (44, 48, 53, 54). In lymphoid organs, Tfr cells inhibit B cell activation, class switch recombination, and somatic hypermutation while also restraining Tfh cell cytokine production (17, 20, 55). Tfr deficiency results in abnormal GC responses paired with increased autoantibody production and autoimmunity (21, 55, 56). Further studies are needed to define the functional capacity of Tph-like Tregs and to confirm their role in T-B cell interactions in the arthritic joint.

The transcriptional program leveraged by Tph and Tph-like Tregs to enable their effector functions in peripheral tissues is not fully understood. Our combined scRNA-seq dataset highlights transcriptional regulators that may be involved in determining Tph and Tph-like Treg function, notably *BHLHE40* (34, 45). BHLHE40 is a basic helix-loop-helix transcription factor that has been associated with cytokine production in CD4^+^ T cells (57, 58). In CD8^+^ tumor-infiltrating lymphocytes, *BHLHE40* promotes tissue residency and cytokine polyfunctionality by controlling the metabolic and epigenetic state of these cells (59, 60). This transcription factor may exert a similar role in Tph and Tph-like Tregs and enable these cells to reside in tissues. *ID2* transcripts are also markedly increased in both Tph and Tph-like Tregs found in the joint. Id proteins inhibit helix-loop-helix DNA-binding transcription factors, raising the possibility that ID2 may regulate BHLHE40 (61). In mice, Id2 selectively downregulates the lymph node trafficking receptor *Cxcr5* in CD4^+^ T cells (62). Similarly, *Id2* and *Id3* expression in mouse Tregs is required to decrease surface Cxcr5 levels to allow Tregs to traffic to peripheral tissues and control inflammation at mucosal sites (63, 64). In sum, both *BHLHE40* and *ID2* emerge as important transcriptional regulators that may allow Tph and Tph-like Tregs to maintain their tissue residency and function. In addition, *ALOX5AP* was highly upregulated in both Tph and Tph-like Tregs compared to other T cell populations in the joint, suggesting that the leukotriene pathway may also be important for the function of B cell-helper T cells in tissues.

Our findings also have important implications for oligo JIA, which is the most common form of chronic inflammatory arthritis in children living in North America (65). Prior studies demonstrated enrichment of PD1^hi^ and IL-21^+^ CD4 T cells in the SF of ANA^+^ oligo JIA patients (35, 41). We extended upon these findings by evaluating other markers associated with B cell help, including CXCL13 and ICOS. Consistent with previous work, Tph enrichment in the JIA joint is associated with ANA positivity. We also find significantly increased frequencies of CXCL13^+^ Tph cells within SF samples obtained from patients diagnosed with oligo JIA before 6 years of age, consistent with the coalescence of ANA positivity and early disease onset in some patients (26–28). While previous studies of PD1^hi^ CD4^+^ T cells in oligo JIA focused solely on protein expression, our scRNA-seq studies highlighted substantial differences in the transcriptional activity of Tph cells found in ANA^-^ or ANA^+^ samples, with a marked upregulation of the Tph transcriptomic signature in ANA^+^ SF Tph. It should be noted that Tph cells were observed in ANA^-^ patients, albeit at lower proportions than autoantibody-positive children. In addition to increased frequencies of Tph cells in the joint, Tph cells may be functionally different in ANA^+^ vs. ANA^-^ oligo JIA, which our transcriptomic data suggests. It is also possible that ANA status measured in peripheral blood does not capture all patients with Tph-driven JIA. Other features such as young age at disease onset or detection of autoantibodies in the joint, where Tph cells are active, may be more accurate surrogates. While these points will need to be explored, our current findings identify quantitative and qualitative differences in Tph cells found in the joints of ANA^+^ and ANA^-^ oligo JIA patients and support the hypothesis that dysregulated T cell help to B cells drives disease in ANA^+^ oligo JIA.

This research was conducted with samples from patients with oligo JIA and has limitations associated with human-based research. While stimulation with CD3/CD28-coated beads is preferred to detect CXCL13, T cells responses to this type of stimulation can vary depending on the prior levels of activation in the source sample, which introduces inter-sample variability in the measurement of cell markers. Similarly, the variability from subject to subject and occasional high rates of lymphocyte cell death in our *in vitro* studies reduced the sensitivity of these assays as a readout for T cell-help to B cells. We attempted to control for this limitation by applying strict and objective quality control criteria for inclusion of an experiment in our analyses. Our single-cell studies combine data from two distinct experiments and laboratories, which strengthens the generalizability of the findings but also introduces data-related challenges. To account for this, we employed careful normalization and integration techniques for the combined analysis of single-cell data from distinct 10X chips, including use of the Harmony tool (66). The elimination of clustering by technical batch in our re-clustering analysis supports the validity of our approach. Our studies report on protein and gene expression in Tph-like Tregs but the function or dysfunction of this Treg subset in inflamed tissues remains to be defined. The study of this Treg population in the context of autoimmune diseases may be difficult to interpret and needs to be evaluated in other human or animal models.

Our study provides insights into the important role of Tph cells in the inflamed tissues of patients with autoantibody-positive arthritis. A subset of Treg cells was identified, with features characteristic of T cells that can regulate the GC reaction and the generation of autoantibodies. Through single-cell gene expression studies, we uncovered novel transcriptional regulators that may be important in enabling Tph and Tph-like Tregs to reside and function in peripheral tissues. In total, interactions among Tph-B-Treg cells likely shape autoantibody-mediated inflammation in tissues, and the function and regulation of this axis warrants further study.

## 4 Materials and methods

### 4.1 Study cohort

Patients with oligo JIA defined by the ILAR criteria were recruited from the Rheumatology Clinic BCH (29). Medical record review was conducted by a board-certified pediatric rheumatologist to confirm eligibility and collect relevant clinical information. Patients with a positive readout for ANA at any timepoint and titer throughout their disease course were categorized as ANA^+^. Synovial fluid and/PB samples obtained within 6 months of symptom onset as reported by the patient were considered as new-onset samples. Medical records were reviewed closer to the time of publication to determine the disease course (persistent or extended oligo JIA). **Table 1** and **Supplemental Table S1** detail the patients and samples included in this study.

### 4.2 Sample processing

PB and SF samples from oligo JIA patients were collected at the same time as a clinically indicated blood draw or joint procedure. Children seen in the Rheumatology Clinic at BCH for non-inflammatory causes of joint pain were recruited as pediatric controls. Healthy adults also volunteered to provide PB samples. Peripheral blood was collected in tubes with ethylenediaminetetraacetic (EDTA). In addition, discard samples were obtained from heathy adults who donated blood to the BCH Blood Conation Center as leukopaks. In all cases, Ficoll density gradient centrifugation (GE Healthcare, now Cytiva) was used to isolate mononuclear cells (MCs). Resulting PBMCs and SFMCs were used fresh or cryopreserved in liquid nitrogen before thaw, as indicated for each application.

### 4.3 Flow cytometry

For flow cytometry analyses of CD4^+^ T cells, samples were thawed and washed in PBS (HyClone) and resuspended into complete media (RPMI (HyClone) with 10% FBS (Genesee Scientific), penicillin 100 IU/mL (Corning Cellgro), streptomycin 100 μL/mL (Corning Cellgro), nonessential amino acids (Corning Cellgro), sodium pyruvate 1mM (Corning Cellgro), and HEPES 10mM (ThermoFisher Scientific)) for overnight incubation. For Tph quantification, CXCL13 and ICOS staining, cells were incubated with CD3/CD28 Dynabeads (Gibco) for 18 to 20 hours, in the presence of brefeldin A (GolgiPlug, BD Biosciences) in the last 4 hours. For HLA-DR and cytokines staining, cells were rested overnight and stimulated with a PMA, ionomycin and brefeldin A cocktail (BD Biosciences) for 4 hours. For all samples and panels, some cells were also left unstimulated as a control condition. Cells were harvested in PBS, incubated in the presence of Live/Dead Fixable Yellow dye (Invitrogen) for 20 min at 4°C, and incubated with surface antibody markers in FACS buffer (PBS with 1.5% FBS (Genesee Scientific) and 2.5 mM EDTA (Invitrogen)) for 40 min at 4°C. Cells were then fixed and permeabilized per the FOXP3 staining kit protocol (eBioscience), before staining for intracellular markers for 30 min at 4°C.

For flow cytometry analyses of CD19^+^ B cells, total SFMCs or PBMCs were thawed as detailed above and directly stained for surface markers. Alternately, the CD4-depleted fraction of samples processed for functional assays was used. Antibodies used in flow cytometry studies are listed in **Table S2**. All flow cytometry data were acquired immediately on an LSRFortessa (BD Biosciences) and analyzed in FlowJo.

### 4.4 B cell help functional assays

Cryopreserved SFMCs were thawed in plain RPMI (HyClone) and processed immediately for CD4^+^ T cell enrichment through negative selection (Miltenyi Biotec). Tfh (CD4^+^CD45RA^-^PD-1^+^CXCR5^+^), Tph (CD4^+^CD45RA^-^PD-1^hi^CXCR5^-^) and double-negative (CD4^+^CD45RA^-^PD-1^-^CXCR5^-^) cells were FACS-sorted on a FACS Aria II. PBMCs from third-party donors were freshly isolated from leukopaks, processed for CD19^+^ cell enrichment through positive selection (Miltenyi Biotec), and then memory B cells (CD3^-^CD19^+^IgD^-^CD27^+^) cells were isolated by FACS.

Sorted T and B cells were co-cultured in complete media (contents as described above) at a ratio of 1 T cell to 10 B cells and in the presence of staphylococcal enterotoxin B (SEB, Fisher Scientific LLC) at a concentration of 1 μg/mL. After 5 days of incubation, culture supernatants were collected for assessment of total IgG production with an ELISA kit (ThermoFisher Scientific) and cells were stained for assessment of viability (7-AAD viability dye) and plasmablast differentiation (CD19^+^CD27^+^CD38^hi^). To be considered in the final analysis, co-culture readouts had to meet the following criteria, suggestive of a successful co-culture with sufficient cell viability: greater than 10% lymphocytes and visible lymphocyte proliferation in FSC/SSC gate, greater than 40% live B cells in CD19/Live-Dead gate, and at least one control population meeting the previous criteria for the concerned experiment.

### 4.5 Single-cell transcriptomics analyses

Single-cell data from 2 previously published studies (34, 45) were integrated and re-analyzed in R version 4.1.0 using Seurat version 4.0.3 (67, 68). Both studies initially used 10X Genomics to analyze FACS-sorted Treg (CD3^+^CD4^+^CD25^+^CD127^lo^) and Teff (CD3^+^CD4^+^CD25^-^) cells from JIA SF. Maschmeyer et al. further sorted those populations based on expression of CD45RO, which is expressed on the vast majority of CD4^+^ T cells in oligo JIA SF (34). One patient with disease onset at 17.5 years did not meet the ILAR criteria for oligo JIA and was therefore excluded from this re-analysis.

Teff and Treg transcriptomic data were re-analyzed independently. For each T cell population, relevant filtered matrices of raw features, as generated with CellRanger in the initial studies, were imported in R using Seurat’s Read10X() function. Transcriptomic data from all runs were combined into a single dataset for each T cell population, and re-examined for quality control. Droplets with <2000 unique molecular identifier (UMIs), <1000 genes, a log10 of genes per UMI <0.8, or a mitochondrial to nuclear gene ratio >0.1 were excluded from the Teff dataset; and those with <2400 UMIs, <1200 genes, <0.8 log10 genes per UMI, or >0.1 mitochondrial to nuclear gene ratio were excluded from the Treg dataset. Further, cells that did not express *CD3E*, representing <2% of each dataset, were excluded. Transcriptomic data were then normalized for each 10X run using Seurat SCTransform() while regressing out the effect of the number of UMIs, number of genes and ratio of mitochondrial to nuclear transcripts per cell. Finally, normalized data were integrated across runs using the standard Seurat integration workflow and exclusion of *TRAV*, *TRAJ*, *TRBV* and *TRBJ* genes from the integration anchors to avoid clustering by clonotype (67, 68).

The first 40 principal components (PCs) were calculated using Seurat’s RunPCA() function and used to define the uniform manifold approximation and projection (UMAP) coordinates. Louvain clustering analysis was run on integrated data using the first 30 (Treg dataset) to 40 PCs (Teff) and following the standard Seurat clustering analysis workflow (67, 68). We opted for a resolution of 0.4 (Teff) to 0.6 (Treg) to delineate the clusters. Cluster identities were determined by running Seurat’s FindAllMarkers() using the default Wilcoxon rank sum test to identify key markers of each cluster. A contaminant cluster of Treg cells (expressing *FOXP3* and clustering apart on the UMAP) was identified in the Teff data and excluded from downstream analyses.

Cells were scored for the Tph, Tfh and/or induced Treg (iTreg) signatures using Seurat’s AddModuleScore() (44, 47, 69). Differential gene expression analyses (DEA) of Tph and Tph-like Tregs were run using Seurat’s FindMarkers() with the receiving operating curve (ROC) classifier method and assessing genes expressed in >5% of the cells in either of the contrasted groups. For DEA, the clusters of mitotic cells were excluded from the contrast group. Ranked log2 fold change (LFC) values were used as input for gene set enrichment analyses (GSEA) using clusterProfiler v4.0.2 and querying the Reactome database (70, 71).

To contrast the Tph-like Tregs (identified in the Treg dataset) with Teff cells, the quality-controlled Treg and Teff datasets were eventually combined, normalized and re-integrated across patients as described above. A DEA of Tph-like Tregs versus Teff (excluding mitotic cells) was run using Seurat’s FindMarkers() function with the Wilcoxon rank sum test. Gene lists resulting from all marker identification and differential gene expression analyses are provided in **Supplemental File S1**.

### 4.6 Single-cell TCR repertoire analyses

For TCR repertoire data derived from 10X studies of SF samples, only the subset of cells included in transcriptomic analyses was considered to enable matching of each clonotype to its cluster identity. For PB samples, the complete TCR repertoire data as compiled by CellRanger vdj was considered. In both cases, clonotypes were defined by their paired CDR3 alpha and beta amino acid sequences and *TRAV*, *TRAJ*, *TRBV* and *TRBJ* genes. The percentage of overlap between 2 repertoires from different clusters or compartments (PB and SF) was obtained by averaging the fractions of unique overlapping clonotypes among all unique clonotypes in each of the 2 repertoires.

### 4.7 Statistics

One-way ANOVA with Turkey correction or two-way ANOVA with Šídák correction for multiple testing were used to assess for differences across >2 groups, and two-tailed Student’s t-test were used to assess for differences between exactly 2 groups. A P-value of <0.05 was considered significant. All statistical analyses were run in GraphPad Prism v9.2.0.

### 4.8 Study approval

This study was conducted in accordance with IRB protocols 07-09-0375 and P00005723. Written informed consent (and assent when appropriate) was obtained for the study participants.

## Data availability statement

Publicly available datasets were analyzed in this study. The datasets can be found here: 1)https://www.immport.org under study accession SDY1777 and 2) the Gene expression omnibus (GEO) under the accession number GSE160097.

## Ethics statement

The studies involving human participants were reviewed and approved by Boston Children’s Hospital Institutional Review Board. Written informed consent to participate in this study was provided by the participants’ legal guardian/next of kin.

## Author contributions

LH and AJ conceived and designed the study. LH supervised the research. DR contributed to the experimental design. AJ, KL, MT, and KH, performed the experiments and acquired the data. PL conducted the ELISA assays. AJ conducted the scRNA-seq experiments and analysis with input from KW and MG-A. PS provided gene signatures for the scRNA-seq analysis. MT, KH, SC, MC, MHC, EC, FD, OH, JH, MH, EJ, JL, ML, EM, JR, HW, MS, RS, PL, PN and LH recruited patients and collected samples. AJ, KL, and LH analyzed the data. AJ and LH drafted the manuscript with input from DR and PN. All authors edited the manuscript and approved the final version.
