# Supplementary material for: Disordered T cell-B cell interactions in autoantibody-positive inflammatory arthritis
Source: Front Immunol. 2023 Jan 5;13:1068399. doi: 10.3389/fimmu.2022.1068399 (PMC9849554; doi:10.3389/fimmu.2022.1068399)
Supplement: Supplementary file 1 [file DataSheet_1.pdf]

Supplementary Material

1 Supplementary Data

1.1 Supplemental File 1. Single cell transcriptomic analyses

2 Supplementary Figures

2.1 Supplementary Figure 1. Flow cytometry gating strategy for T cell populations.

(A) Representative dot plots depicting the flow cytometry gating strategy for Tph cells in oligo JIA PB and SF. **(B)** Representative flow cytometry dot plots depicting CXCR5 gating in Tph cells and Tregs from the SF of a patient with oligo JIA and PB of an adult control. CXCR5 gating was determined with the use of an FMO. **(C)** Representative dot plots depicting the flow cytometry gating strategy for Tph-like Tregs in the PB and SF of patients with oligo JIA. This sample was stimulated with CD3/CD28 Dynabeads to evaluate subsequent CXCL13 expression (Figure S2). Oligo JIA, oligoarticular juvenile idiopathic arthritis; PB, peripheral blood; SF, synovial fluid; Tph, T peripheral helper cell; FMO, fluorescence minus one;

2.2 Supplementary Figure 2. CXCL13 and ICOS expression in oligo JIA T cells.

(A) Representative dot plots gated on live $CD3^+CD4^+$ T cells from oligo JIA PB and SF. **(B)** Representative dot plots gated on live Tregs from oligo JIA PB and SF. Oligo JIA, oligoarticular juvenile idiopathic arthritis; PB, peripheral blood; SF, synovial fluid.

2.3 Supplementary Figure 3. Flow cytometry gating strategy for B cell populations.

(A) Representative dot plots depicting the flow cytometry gating strategy for B cells in oligo JIA SF and adult control PB **(B)** Representative dot plots depicting the flow cytometry gating strategy for the read out from a T cell-B cell co-culture. Oligo JIA, oligoarticular juvenile idiopathic arthritis; PB, peripheral blood; SF, synovial fluid.

2.4 Supplementary Figure 4. Single-cell transcriptomics studies of oligo JIA synovial fluid Teffs and Tregs.

Combined data from two previously published datasets of oligo JIA SF Teff ($CD4^+CD25^-$) and Treg ($CD4^+CD127^{lo}CD25^+$) cells studied with 10X Genomics.(34, 43) **(A)** UMAP of SF Teffs from 8 oligo JIA patients, split by ANA subgroup and color-coded by cluster. **(B)** Frequency of each cluster among total SF Teffs for each patient, contrasted by ANA subgroup. **(C)** UMAP of SF Teffs split between ANA^- (9667 cells) and ANA^+ (7769 cells) groups, color-coded with their Tfh signature score.(42) **(D)** Top pathways identified in geneset enrichment analysis (GSEA) of SF Tph (Clusters 4 and 5) versus other SF Teffs (Clusters 1-3 and 6-10), ranked by normalized enrichment score (NES). **(E)** UMAP of SF Tregs split between ANA^- (4550 cells) and ANA^+ (7606 cells) groups, color-coded with their induced Treg (iTreg) signature score.(68) SF, synovial fluid; ANA, antinuclear autoantibody; Teff, T effector cell; Tph, T peripheral helper cell; Tfh, T follicular helper cell; Treg, T regulatory cell; oligo JIA; oligoarticular juvenile idiopathic arthritis; UMAP, uniform manifold approximation and projection; TCR, T cell receptor.

3 Supplementary Tables

3.1 Table S1. Clinical characteristics of the study participants and samples contributed to the studies.

Subject	Sample	Group	Oligo JIA subtype		Sex	Age (years)		Joint involvement		ANA	Uveitis	Treatment	Flow cytometry		Co-culture
			at sampling	end of study		at onset	at sampling	at onset	at sampling			at sampling	CD4	CD19	
Adult 1•	PB	Control	NA	NA	unk.	NA	unknown	NA	NA	NA	NA	NA	No	Yes	NA
Adult 2•	PB	Control	NA	NA	unk.	NA	unknown	NA	NA	NA	NA	NA	No	Yes	NA
Adult 3	PB	Control	NA	NA	F	NA	22	NA	NA	NA	NA	NA	Yes	Yes	NA
Adult 4	PB	Control	NA	NA	F	NA	24-25	NA	NA	NA	NA	NA	Yes	No	NA
Adult 5	PB	Control	NA	NA	M	NA	35-36	NA	NA	NA	NA	NA	Yes	Yes	NA
Adult 6	PB	Control	NA	NA	M	NA	28	NA	NA	NA	NA	NA	Yes	No	NA
Adult 7	PB	Control	NA	NA	F	NA	27-28	NA	NA	NA	NA	NA	No	Yes	NA
Adult 8	PB	Control	NA	NA	F	NA	36-37	NA	NA	NA	NA	NA	Yes	No	NA
Adult 9	PB	Control	NA	NA	F	NA	28-29	NA	NA	NA	NA	NA	Yes	No	NA
Adult 10	PB	Control	NA	NA	M	NA	30-31	NA	NA	NA	NA	NA	Yes	No	NA
Adult 11	PB	Control	NA	NA	M	NA	36-37	NA	NA	NA	NA	NA	No	Yes	NA
Pedi 1	PB	Control	NA	NA	F	NA	11.5	NA	NA	NA	NA	NA	Yes	No	NA
Pedi 2	PB	Control	NA	NA	M	NA	11	NA	NA	NA	NA	NA	Yes	No	NA
Pedi 3	PB	Control	NA	NA	F	NA	6	NA	NA	NA	NA	NA	Yes	No	NA
Pedi 4	PB	Control	NA	NA	M	NA	6.5	NA	NA	NA	NA	NA	Yes	No	NA
Pedi 5	PB	Control	NA	NA	M	NA	7.5	NA	NA	NA	NA	NA	Yes	No	NA
JIA01*	SF	Oligo	persistent	persistent	F	4.5	9	2	1	-	Yes	ADA	No	No	No

JIA02*	SF	Oligo	persistent	persistent	F	2	4	1	1	+	No	ETA	No	Yes	Yes
JIA03	SF	Oligo	persistent	persistent	F	3.5	9	1	1	-	No	None	Yes	No	No
JIA04	SF	Oligo	persistent	persistent	F	10	13	1	1	+	No	MTX	Yes	No	No
JIA04b	SF	Oligo	persistent	persistent	F	10	16	1	1	+	No	MTX	Yes	Yes	Yes
JIA05	PB, SF	Oligo	persistent	persistent	F	5	9.5	1	1	-	No	LEF	Yes	No	No
JIA06	SF	Oligo	new-onset	persistent	F	2.5	2.5	2	4	+	No	None	No	Yes	Yes
JIA07	SF	Oligo	persistent	persistent	F	5.5	6	1	1	+	No	None	No	Yes	No
JIA08	PB, SF	Oligo	new-onset	persistent	M	2	2.5	3	3	+	No	None	Yes	No	No
JIA09	PB, SF	Oligo	new-onset	persistent	F	4.5	4.5	3	3	-	No	None	Yes	Yes	No
JIA10	PB, SF	Oligo	new-onset	persistent	F	5	5	2	2	-	No	None	Yes	No	No
JIA11	PB, SF	Oligo	new-onset	unknown	F	11	11.5	1	1	-	No	None	Yes	Yes	No
JIA12	PB, SF	Oligo	new-onset	persistent	F	3	3	1	1	+	No	None	Yes	No	No
JIA13	PB, SF	Oligo	new-onset	persistent	F	7.5	7.5	2	2	-	No	None	Yes	Yes	Yes
JIA14	PB, SF	Oligo	new-onset	persistent	M	4	4	1	1	-	No	None	Yes	No	No
JIA15	PB, SF	Oligo	new-onset	persistent	F	7.5	7.5	2	2	+	No	None	Yes	No	No
JIA16	PB, SF	Oligo	persistent	persistent	F	4	13	1	2	+	No	None	Yes	Yes	No
JIA17	SF	Oligo	extended	extended	F	6	14.5	1	1	-	No	None	Yes	Yes	No
JIA18	SF	Oligo	persistent	persistent	M	6	6.5	1	2	-	No	MTX	Yes	No	No
JIA19	SF	Oligo	persistent	persistent	F	6	17	3	2	+	No	None	Yes	No	No
JIA20	SF	Oligo	extended	extended	F	7	20	1	1	+	No	ADA	Yes	No	No
JIA21	SF	Oligo	extended	extended	F	4	15	4	1	+	Yes	None	Yes	Yes	No
JIA22	SF	Oligo	persistent	persistent	F	6	11.5	1	2	-	No	None	No	Yes	No
JIA23	SF	Oligo	persistent	persistent	F	2	8	1	2	-	Yes	None	Yes	Yes	Yes
JIA24	PB, SF	Oligo	persistent	persistent	F	2	2.5	2	3	+	No	MTX	Yes	No	No
JIA25	SF	Oligo	new-onset	undiff.	F	13	13	1	2	+	No	None	Yes	No	No

JIA26	SF	Oligo	persistent	extended	F	2.5	10	1	1	-	No	None	Yes	No	No
JIA26b	SF	Oligo	persistent	extended	F	2.5	14.5	1	1	-	No	ETA	Yes	Yes	No
JIA27	SF	Oligo	persistent	persistent	F	3	9	2	2	+	No	MTX	Yes	Yes	No
JIA28	SF	Oligo	persistent	persistent	F	3.5	10	1	1	-	Yes	MTX	Yes	No	No
JIA29	PB, SF	Oligo	persistent	persistent	F	4.5	8.5	1	2	+	No	None	Yes	No	No

•Leukopaks were obtained from 2 adult controls who donated to the blood bank at Boston Children's Hospital. Per IRB policy, clinical characteristics of these patients (including age and sex) are not shared.

*Sample included in prior 10X studies (see Julé et al., 2021) and considered in the present re-analysis.

NA, not applicable; PB, peripheral blood; SF, synovial fluid; Oligo, oligoarticular; JIA, juvenile idiopathic arthritis; F, female; M, male; ADA, adalimumab; ETA, etanercept; LEF, leflunomide; MTX, methotrexate; ANA, autonuclear antibodies; Undif, undifferentiated; unk, unknow

3.2 Table S2. Antibodies and dyes used in flow cytometry and functional studies.

Antibody or dye	Clone	Manufacturer	Reference	Staining	Panel
anti-CD3-Alexa Fluor 700	HIT3a	BioLegend	300324	Surface	T cell (1) & (2)
anti-CD3-FITC	OKT3	BioLegend	317306	Surface	Sort
anti-CD4-FITC	RPA-T4	BD Biosciences	555346	Surface	T cell (1) & (2)
anti-CD4-PE/Cy7	OKT4	BioLegend	317414	Surface	Functional assay (sort)
anti-CD45RA-FITC	HI100	BioLegend	304106	Surface	Functional assay (sort)
anti-PD-1-APC	EH12.2H7	BioLegend	329907	Surface	T cell (1) & (2), Sort
anti-CXCR5-PerCP/Cy5.5	J252D4	BioLegend	356910	Surface	T cell (1) & (2)
anti-CXCR5-PE/eFluor610	MU5UBEE	eBioscience	61-9185-42	Surface	Functional assay (sort)
anti-CD127-PE/Cy7	A019D5	BioLegend	351320	Surface	T cell (1)
anti-CD25-BV605	BC96	eBioscience	302632	Surface	T cell (1)
anti-HLA-DR-BV605	G46-6	BD Biosciences	562844	Surface	T cell (2)
anti-ICOS-APC/Cy7	ISA-3	eBioscience	47-9948-42	Surface	T cell (1)
anti-CD38-FITC	HB-7	BioLegend	356610	Surface	B cell
anti-CD21-PE	HB5	eBioscience	12-0219-42	Surface	B cell
anti-CD27-APC	O323	BioLegend	302810	Surface	B cell
anti-CD27-APC/Cy7	O323	BioLegend	302816	Surface	Functional assay (sort & readout)
anti-IgD-PerCP/eFluor710	IA6-2	eBioscience	46-9868-42	Surface	B cell, Functional assay (sort & readout)
anti-CD24-PE/Cy7	SN3 A5-2H10	eBioscience	25-0247-42	Surface	B cell
anti-CD19-BV605	HIB19	BioLegend	302244	Surface	B cell, Functional assay (sort & readout)
anti-FOXP3- eFluor450	PCH101	Invitrogen	48-4776-42	Intracellular	T cell (1)
anti-CXCL13-PE	53610	Invitrogen	MA5-23666	Intracellular	T cell (1)
anti-IL-21-PE	3A3-N2	BioLegend	513004	Intracellular	T cell (2)
anti-IFNγ-PE/Cy7	4S.B3	BioLegend	502528	Intracellular	T cell (2)

LIVE/DEAD™ Fixable Yellow Dead Cell Stain	NA	Invitrogen	L34959	Dead cells	T cell (1) & (2)
7-AAD	NA	BioLegend	420404	Dead cells	Functional assay (readout)

The "T cell (1)" panel required overnight stimulation with CD3/CD28-coated beads for detection of CXCL13. For the "T cell (2)" panel, cells were stimulated with PMA/ionomycin for detection of cytokines. See Methods for details.
