# Supplementary figures and images for: Disordered T cell-B cell interactions in autoantibody-positive inflammatory arthritis
Source: Front Immunol. 2023 Jan 5;13:1068399. doi: 10.3389/fimmu.2022.1068399 (PMC9849554; doi:10.3389/fimmu.2022.1068399)

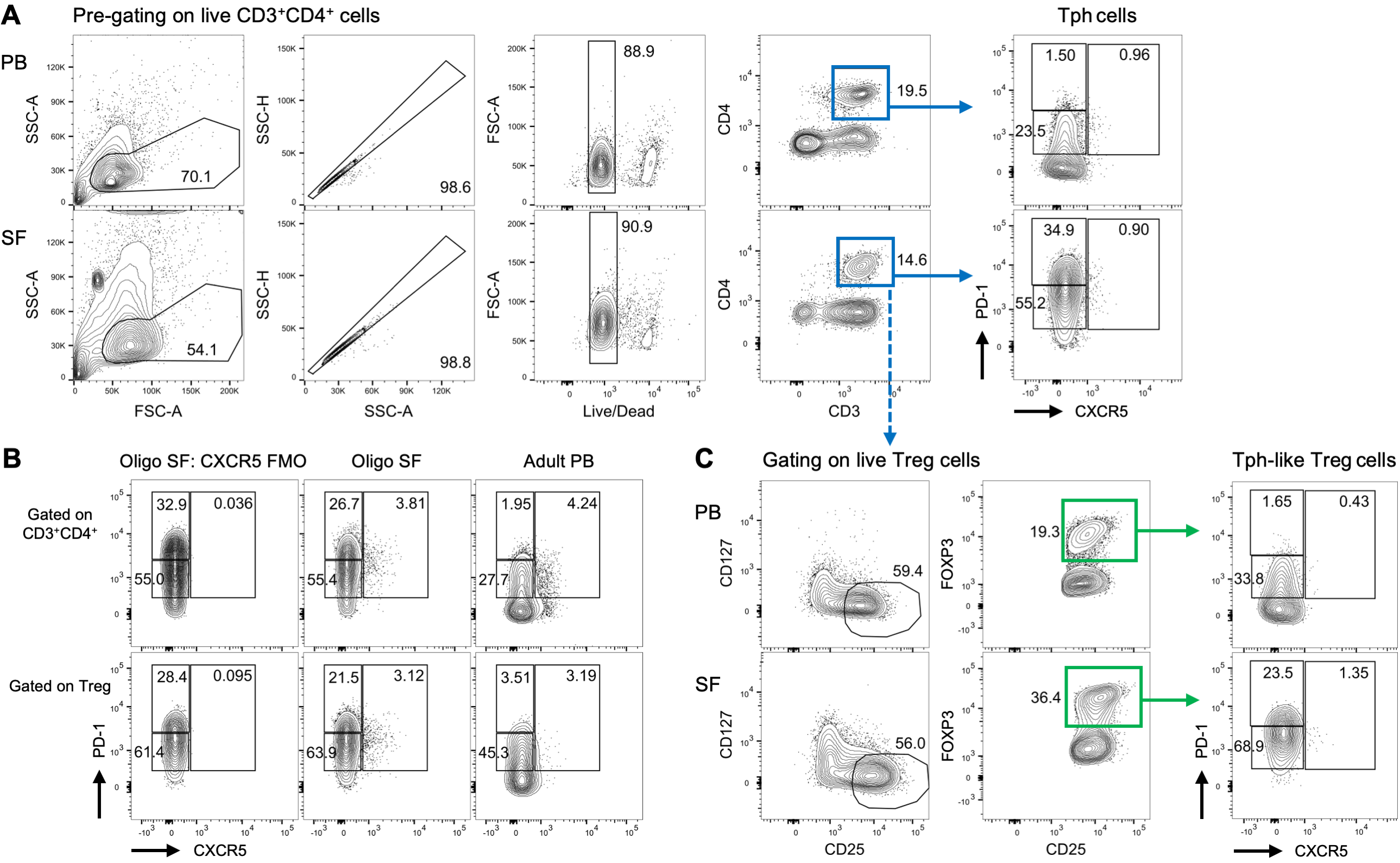

Supplement: Supplementary file 2 [file Image_1.tiff]

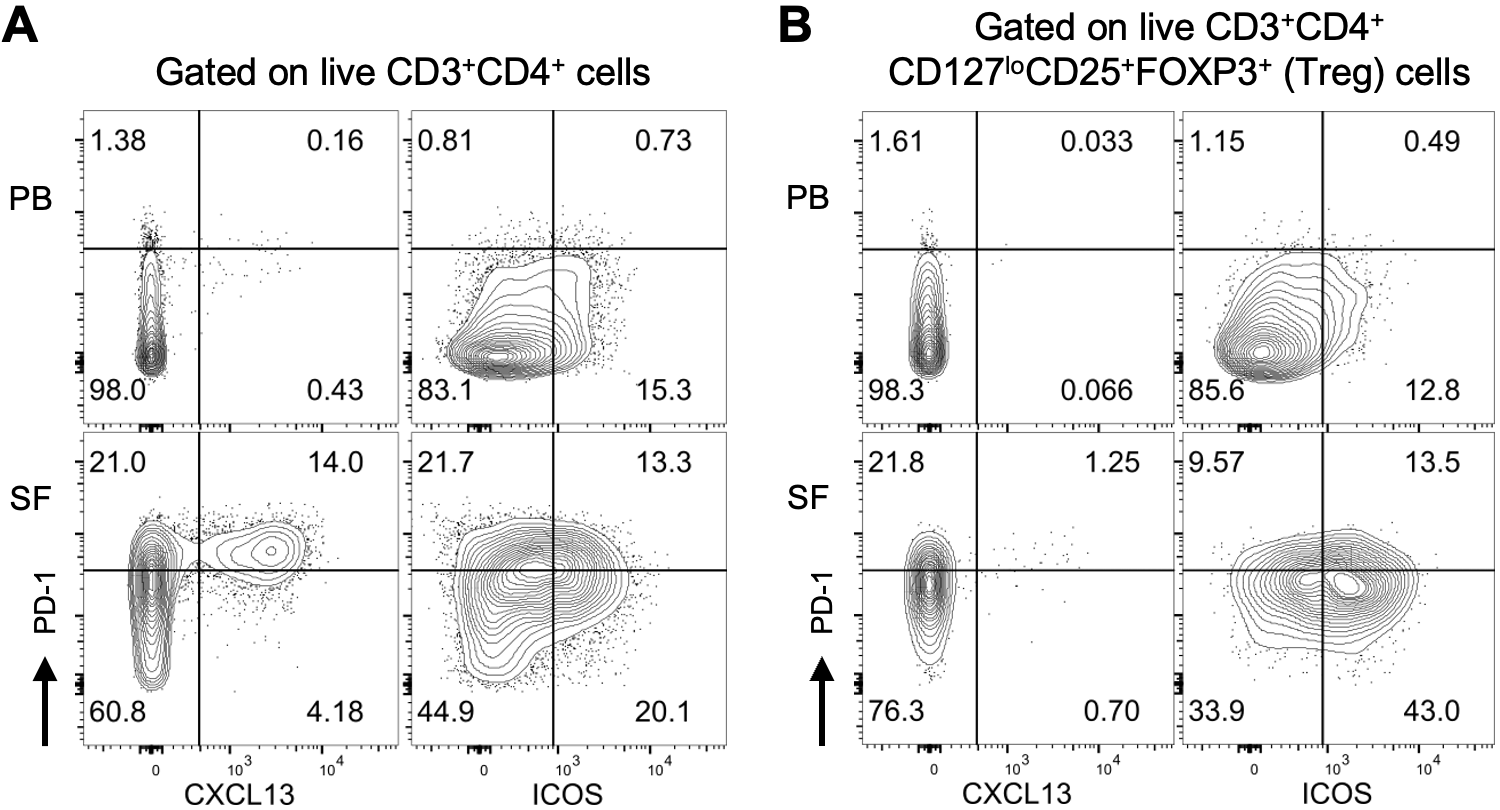

Supplement: Supplementary file 3 [file Image_2.tiff]

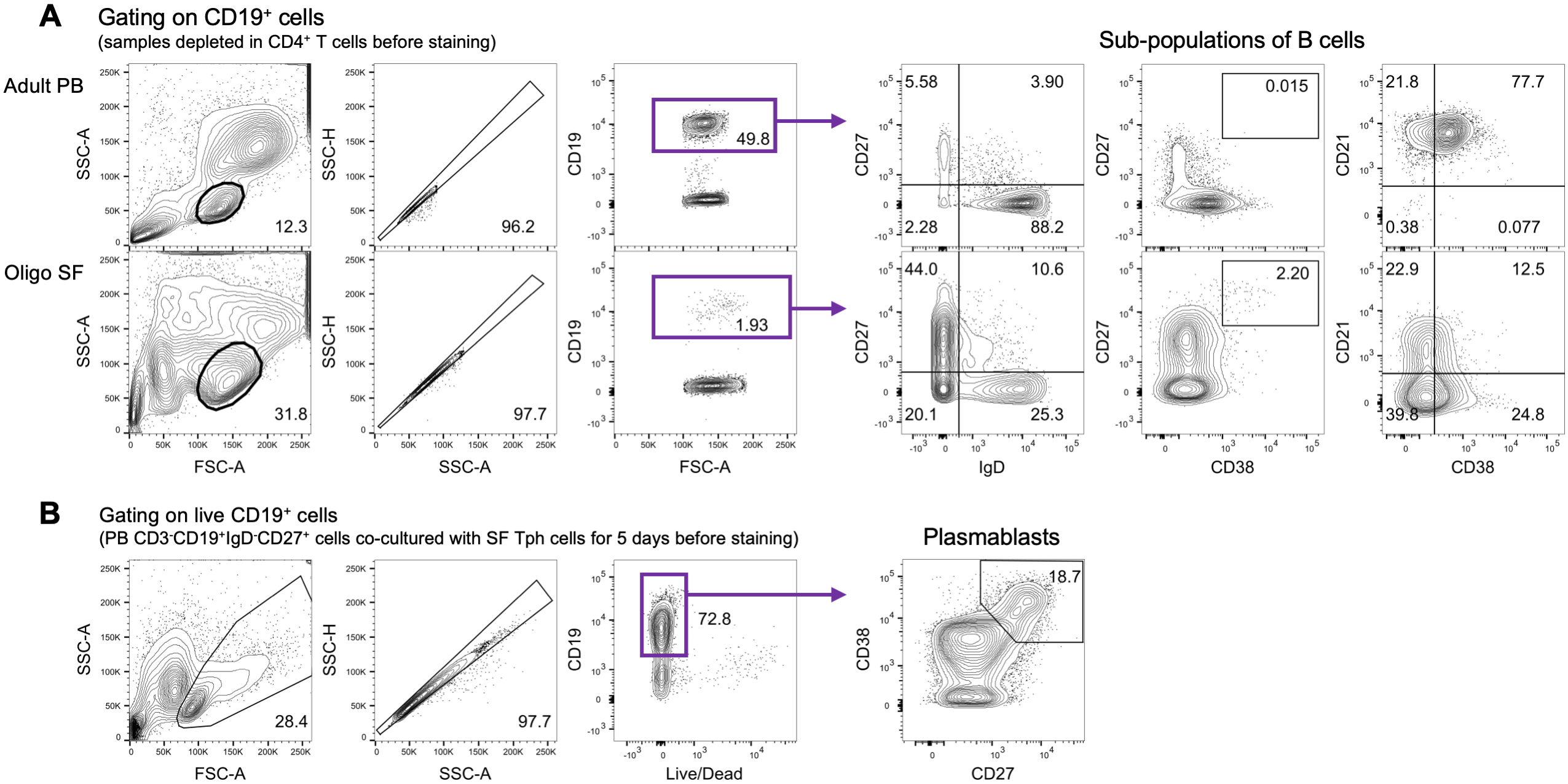

Supplement: Supplementary file 4 [file Image_3.tiff]

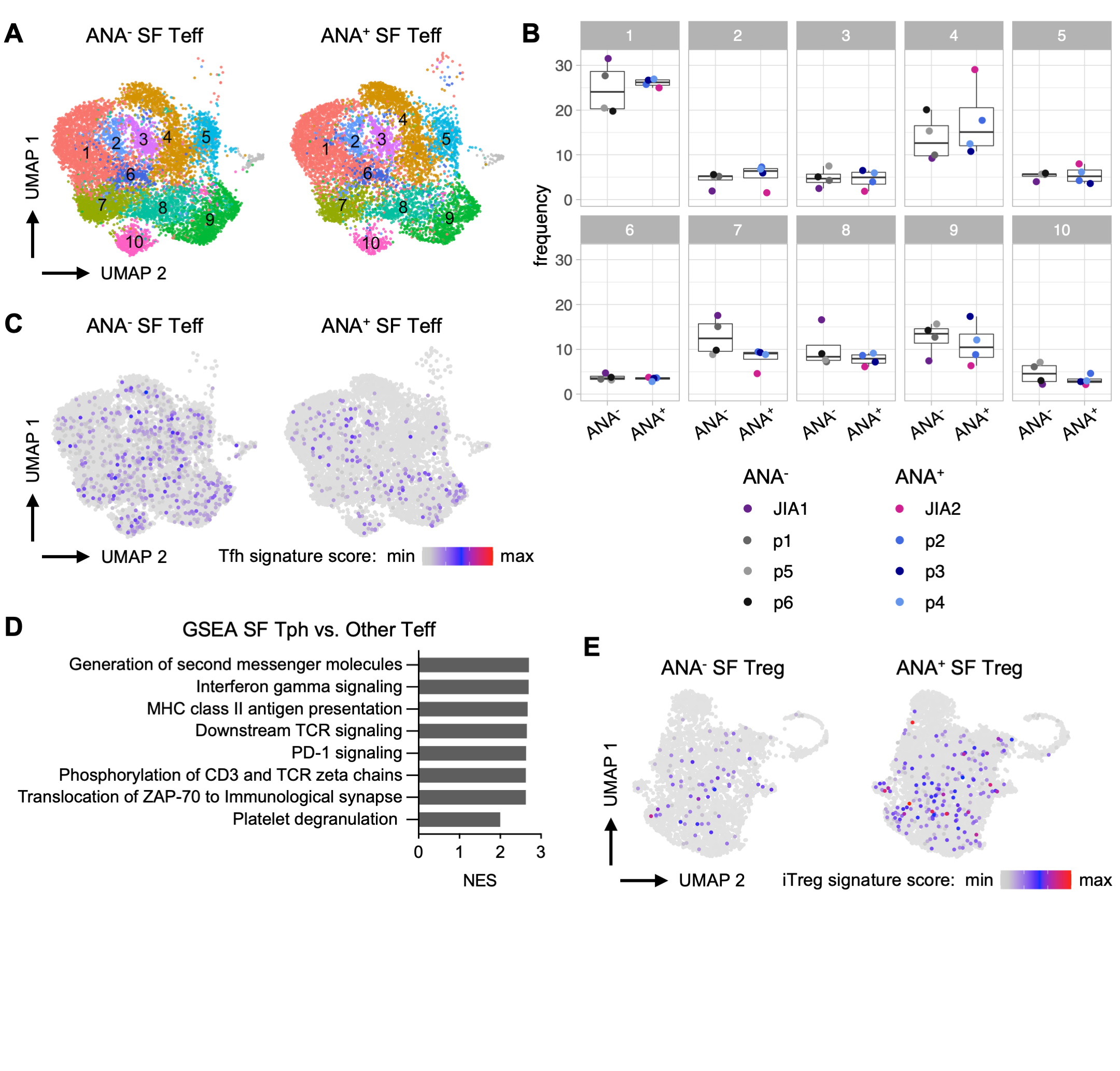

Supplement: Supplementary file 5 [file Image_4.tiff]
